# Structural biology of SARS-CoV-2: open the door for novel therapies

**DOI:** 10.1038/s41392-022-00884-5

**Published:** 2022-01-27

**Authors:** Weizhu Yan, Yanhui Zheng, Xiaotao Zeng, Bin He, Wei Cheng

**Affiliations:** 1 Division of Respiratory and Critical Care Medicine, Respiratory Infection and Intervention Laboratory of Frontiers Science Center for Disease-Related Molecular Network, State Key Laboratory of Biotherapy, West China Hospital of Sichuan University, 610041 Chengdu, China; 2 Department of Emergency Medicine, West China Hospital of Sichuan University, 610041 Chengdu, China; 3 The First People’s Hospital of Longquanyi District Chengdu, 610100 Chengdu, China

**Keywords:** Molecular medicine, Structural biology

## Abstract

Severe Acute Respiratory Syndrome Coronavirus-2 (SARS-CoV-2) is the causative agent of the pandemic disease COVID-19, which is so far without efficacious treatment. The discovery of therapy reagents for treating COVID-19 are urgently needed, and the structures of the potential drug-target proteins in the viral life cycle are particularly important. SARS-CoV-2, a member of the *Orthocoronavirinae* subfamily containing the largest RNA genome, encodes 29 proteins including nonstructural, structural and accessory proteins which are involved in viral adsorption, entry and uncoating, nucleic acid replication and transcription, assembly and release, etc. These proteins individually act as a partner of the replication machinery or involved in forming the complexes with host cellular factors to participate in the essential physiological activities. This review summarizes the representative structures and typically potential therapy agents that target SARS-CoV-2 or some critical proteins for viral pathogenesis, providing insights into the mechanisms underlying viral infection, prevention of infection, and treatment. Indeed, these studies open the door for COVID therapies, leading to ways to prevent and treat COVID-19, especially, treatment of the disease caused by the viral variants are imperative.

## Introduction

**Fig. 1:**
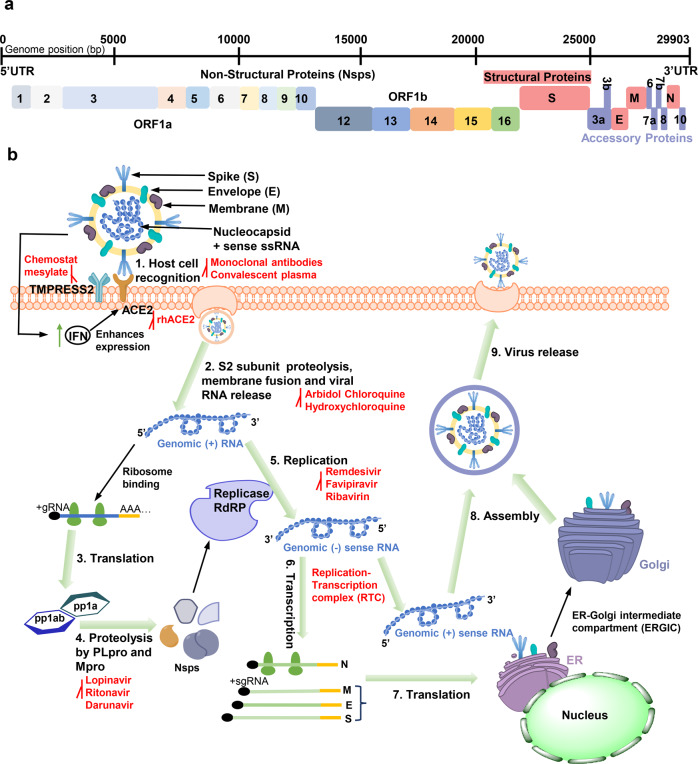
The whole-genome composition and replication cycle of SARS-CoV-2 and potential targets. **a** The viral genome encodes 16 nonstructural proteins (Nsps) required for replication/transcription and structural proteins required for the assembly of new virions. **b** the SARS-CoV-2 mainly infects lymphatic epithelial cells and type II pneumocytes with the initiation of human body’s innate response by producing interferons (IFNs). However, IFN activates expression of ACE2 protein which acts as receptor for virus attachment to host cells. Interaction between S protein and ACE2 leads to proteolytic cleavage at the S1–S2 boundary and S2ʹ site mediated by transmembrane protease serine 2 (TMPRSS2), further inducing the viral and host cell plasma membrane fusion. The single-stranded RNA in the viral genome is translated by host machinery to produce viral polypeptides (pp1a and pp1ab), which undergo proteolytic cleavage by PLpro and Mpro proteins to synthesize Nsps. These Nsps encode replication transcription complex (RTC), which continuously replicates and produces a series of subgenomic messenger RNAs that encode the accessory and structural proteins. The viral genomic RNA and proteins are assembled to form the virus particles in the ER-Golgi intermediate compartment (ERGIC). The vesicle-containing virus then fuses with plasma membrane of the host, releasing the viral particles out of the cell The antiviral molecules with target sites are highlighted in red

SARS-CoV-2, a novel human coronavirus, broke out in December 2019 and has infected more than 230 million people and caused 4.87 million deaths, according to the latest data from World Health Organization (WHO; https://www.who.int/emergencies/diseases/novel-coronavirus-2019). Coronaviruses (CoVs) have the largest genomes of the positive-stranded RNA viruses at 26–32 kb, and are divided into four genera: α-, β-, δ-, and γ-CoVs.^1,2^ SARS-CoV-2 has been identified and classified as lineage B of the genus β-coronavirus,^3^ which also includes severe acute respiratory syndrome coronavirus (SARS-CoV) and Middle East respiratory syndrome coronavirus (MERS-CoV). SARS-CoV-2 shares 79.6 and 96% sequence identity with SARS-CoV and the bat coronavirus RaTG13,^4,5^ respectively. Its genome contains fourteen open reading frames (ORFs), which can be divided into two parts. ORF1a and ORF1ab (Fig. 1a), located in the first two-thirds of the viral genome from the 5′-end, are directly translated into two polyproteins (pp1a and pp1ab) by cellular ribosomes.^6^ Subsequently, the two polyproteins are processed by two viral proteases, papain-like protease (PLpro) and main-protease (Mpro), to produce sixteen nonstructural proteins (Nsps), Nsp1–Nsp16.^7^ Collectively, these constitute the replication-translation complex (RTC).^8^ RNA-dependent RNA Polymerase (RdRp) is required for the expression of the remaining one-third of the genome. Notably, replication of the viral genome is also mediated by RdRp.^9^ Subgenomic RNAs utilize the transcription and translation systems of the host to synthesize four structural proteins: spike (S), membrane (M), envelope (E), and nucleocapsid (N), as well as several accessory proteins (ORF3a, ORF3b, ORF6, ORF7a, ORF7b, ORF8, ORF9b, ORF9c, and ORF10).^10–12^ Finally, RNA and structural proteins are assembled into the mature viral progeny, which are released by exocytosis to further infect the host (Fig. 1b).

The severity of the ongoing COVID-19 pandemic has raised an urgent need to develop antiviral drugs, vaccines, and antibodies. Prophylactic vaccines, which stimulate the host to produce humoral and cell-mediated immune responses, are the primary measure currently used for the prevention of SARS-CoV-2 infection. The type of vaccines available includes the following: (1) inactive or live attenuated whole virus vaccine (US20060039926^13^ and CoronaVac [Sinovac Biotech in China]); (2) nucleic acid vaccines, including DNA and mRNA vaccines, such as ino-4800 and mRNA-1273;^14^ (3) recombinant protein vaccines, including recombinant S protein vaccines, recombinant S protein subunit vaccines,^15^ and virus-like particle vaccines; (4) viral vector vaccines, including replication-incompetent vector vaccines, replication-competent vector vaccines, and inactivated virus vectors such as adenoviral vector vaccine;^16^ and (5) other types of vaccines, such as Bacille Calmette-Guerin (BCG) Vaccines.^17^ Moreover, various potential drugs have been proposed for the treatment of COVID-19. These can be divided into the following groups: (1) chemical medicines, such as nucleoside analogs (chloroquine, hydroxychloroquine, remdesivir, tenofovir, and sofosbuvir);^18,19^ (2) Traditional Chinese medicines, such as Lianhua Qingwen;^20^ and (3) biological agents, including antibodies, vaccines, peptides, oligonucleotides (aptamer, antisense oligonucleotides, small interfering RNAs [siRNAs], RNA interference [RNAi]), interferons,^21^ corticosteroids,^22^ plasma,^23^ and mesenchymal stem cells.^24^

Some efficient vaccines and drugs for emergency use have already been approved. However, the emergence of multiple mutants caused by various competitive processes originating from molecule aspect (such as reading frame shifts, replication errors, etc., of virus itself), organism aspect (such as gene re-edit and recombination induced by host adaptive immune response), and population aspect (such as natural selection),^25^ making it critical to identify alternative targets and develop more broad-spectrum antiviral drugs.^26^ Structural biology can be used to study the pathogenic mechanism of viruses, but also provides theoretical information for drug development and optimization. For this purpose, we summarize the representative structures of SARS-CoV-2 and discuss the development of drugs, antibodies, vaccines, and other therapeutic agents for targeting these proteins or the virus.

## Structural proteins of the virus

### S protein

The transmembrane S protein, protruding from the viral surface,^27^ recognizes the host receptor angiotensin-converting enzyme 2 (ACE2), to mediate coronavirus entry into host cells.^28^ S protein remains the main target of many anti-coronavirus drugs, including neutralizing monoclonal antibodies (mAbs), vaccines, and other inhibitors. Further study of its structure will contribute to the development of more effective or broad-spectrum antiviral drugs.

**Fig. 2:**
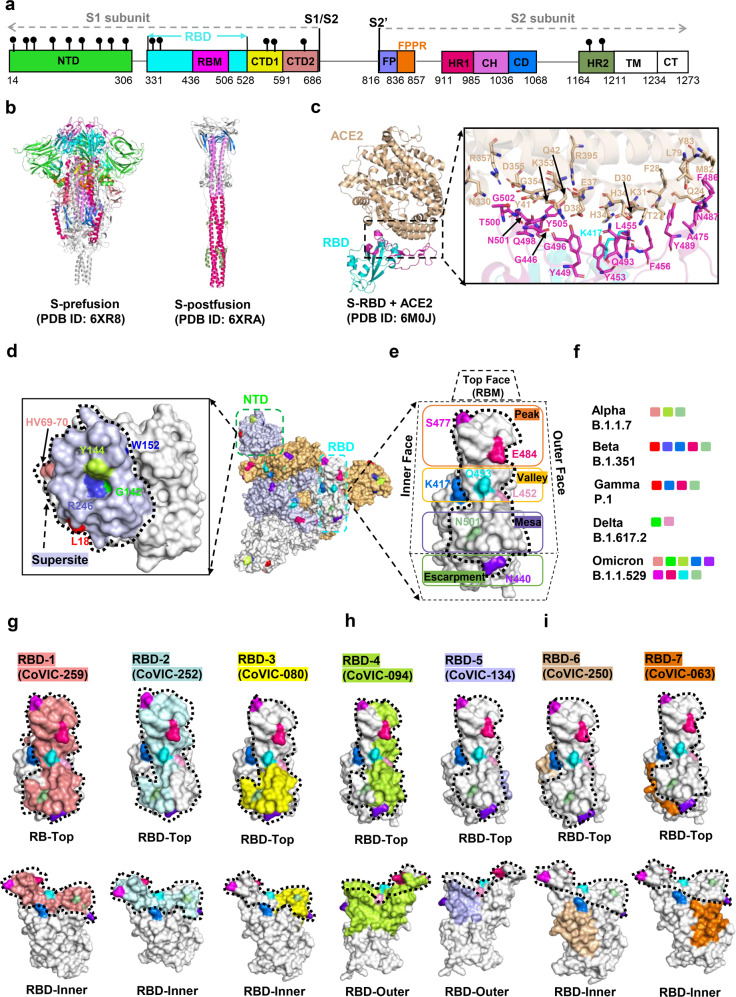
Structures of SARS-CoV-2 Spike protein and antibody recognition. **a** The full-length SARS-CoV-2 S protein. NTD N terminal domain, RBD receptor-binding domain, RBM receptor-binding motif, CTD1 C-terminal domain 1, CTD2 C-terminal domain 2, FP fusion peptide, FPPR fusion-peptide proximal region, HR1 heptad repeat 1, CH central helix region, CD connector domain, HR2 heptad repeat 2, TM transmembrane segment, CT cytoplasmic tail. **b** Structures of S trimer in prefusion and postfusion states. Each domain is marked with a color corresponding to **a**. **c** Structure of the RBD (cyan) in complex with ACE2 (pink). Residues involved in interactions between the RBD and ACE2 are shown as sticks. **d** Footprints for NTD-targeted antibodies, with the NTD “supersite” outlined with a dashed line. The residue positions of important mutations and deletions are indicated in the NTD. Table 1 lists mutations represented in each variant. **e** Location of important emerging mutations on the RBD. The RBM can be topologically divided into three subsections: the “peak” that includes residues S477 and E484; the “valley” that includes K417, Q493, and L452; and the “mesa” includes N501. **f** Mutations and deleted residues affecting antibodies activity involved in significant mutants. **g**–**i** footprint of a representative antibody from the Coronavirus Immunotherapeutic Consortium (CoVIC) mapped onto an RBD monomer. The ACE2 binding site is outlined with a dotted line. The website of CoVIC is at https://covic.lji.org/

SARS-CoV-2 S protein is cleaved by host proteases into two subunits, a receptor-binding fragment (S1) and a fusion fragment (S2), during biogenesis or virus assembly. The S1 subunit consists of an N-terminal domain (NTD, residues 14–306), a receptor-binding domain (RBD, residues 331–528) including receptor-binding motif (RBM, residues 436–506), and the C-terminal domains (CTDs) composed of CTD1 (residues 528–591) and CTD2 (residues 592–686). The S2 subunit is divided into the fusion peptide (FP, residues 816–836), fusion-peptide proximal region (FPPR, residues 837–857), heptad repeat 1 (HR1, residues 911–985), central helix (CH, residues 986–1036), connector domain (CD, residues 1037–1068), heptad repeat 2 (HR2, residues 1164–1211), transmembrane segment (TM, residues 1212–1234), and cytoplasmic tail (CT, residues 1235–1273)^29^ (Fig. 2a).

The S1 subunit of the S trimer wraps around the threefold axis, covering the S2 subunit underneath. The NTDs are located at the periphery of the S trimer and make contact with the RBD from the adjacent protomer.^29^ The RBD is located at the apex of the S1 subunit, which is a key region for coronavirus invasion of host cells. Before and after S protein binds to ACE2, the RBD undergoes a hinge-like conformational change that transiently switches between open (or up) and closed (or down) states.^30^ The open state is required for ACE2 engagement, because the RBM is embedded inside the protein during the closed state.^28,30^ In the S-ACE2 complex, four pairs of disulfide bonds (C336–C361, C379–C432, C391–C525, and C480–C488) are involved in stabilizing the RBD structure, and the RBM forms a concave outer surface to accommodate the N-terminal helix of ACE2. There are 13 hydrogen bonds, two salt bridges (between K417 of the RBD and the D30 of ACE2), and several hydrophobic interactions (between F486 of the RBD and L79, M82, and Y83 of ACE2) contributing to ACE2 engagement^28,30^ (Fig. 2b, c). Notably, the salt bridge interactions between K417 (outside the RBM region) of SARS-CoV-2 S protein and D30 of ACE2 is absent in SARS-CoV.^30^ The CTDs, which is mainly consisted of β-structure, also play an essential role in the structural rearrangement of S protein membrane fusion.^29^ The CTD1 and CTD2 wrap around S2 and are adjacent to RBD and NTD, respectively. And the CTD1 is located between the two NTDs and interacts with NTD from another protomer. A special structural element “630 loop”, located near the S1/S2 boundary and structurally inserted the gap between the NTD and CTD1 of the same protomer, appears in the CTD2 and stabilizes the closed conformation of S protein.^29^

The cleavage of the S protein into S1 and S2 occurs before the fusion of the virus and the host cell membrane, which is necessary for the viral entry into the host cell. And the S2 is further cleaved by TMPRSS2 to FP and S2’ and undergo significant conformational changes during the membrane fusion^31,32^ (Fig. 2b). In the prefusion state, the S2 subunit adopts a conformation packed around a three-strand coiled-coil formed by central CH. A nine helix-bundle is assembled by the central coiled-coil and helix structure of the HR1 together with another helix structure formed by residues 758–784,^33^ which likely stabilizing the overall structure of S trimer. The FPPR and FP are connected directly via a longer loop. And the FPPR tucks underneath CTD1 from another protomer and interacts with CTD2 and HR1 of the same protomer. However, the HR2, TM, and CT regions are disordered in most of the S protein structures that have been determined, except for the low-resolution density that can be observed in cryo-ET reconstruction. In the postfusion state, HR1 undergoes a significant refolding transition, and a long central three-strand coiled-coil of ~180 Å is assembled by a continuous α-helix formed by HR1 and CH.^29^ Besides, HR2 partly becomes ordered and packs against the groove of HR1 coiled-coil with the formation of six-helix-bundle (6HB) structure, which contribute to the fusion of virus and cell membrane.^34,35^

### Antibodies, vaccines, and inhibitors targeting the S protein

A range of antiviral agents targeting the S protein has been developed, such as small molecular inhibitors, antibodies, and vaccines. Generally, inhibitors targeting S glycoproteins block virus-membrane fusion by competitively inhibiting the interaction between RBD and ACE2. Such inhibitors include ivermectin^36,37^ and arbidol (umifenovir).^38–40^ Some also target non-RBD regions, such as lipopeptide EK1 (and its derivative, EK1C4),^41^ a pan-coronavirus fusion inhibitor that targets HR1 of the S2 domain and inhibits membrane fusion.

Monoclonal antibodies elicited from memory B cells of vaccinated or SARS-CoV-2-infected individuals are also effective in prophylaxis or treatment of COVID-19. A number of potential antibodies against SARS-CoV-2 have been identified, some of which target the NTD or S2 domain, and a high proportion of antibodies target RBD. According to the distinct epitope landscape of RBD-directed antibodies, these mAbs can be classified into seven communities (RBD-1 to RBD-7), and each community can be further divided into finer clusters and bins.^42^ Nine antibody types including NTD-, RBD-, and S2-directed antibodies and the mechanism of each are described below.

NTD mAbs (class I, such as 4A8,^43^ FC05,^44^ CM25,^45^ and S2M28^46^) primarily targeted a “supersite”(consisted by residues 14–20, 140–158, and 245–264) of the NTD^46^ (Fig. 2d). Their neutralizing activity does not rely on the sterically inhibition of ACE2 binding,^46^ whereas likely block S-mediated virus-cell fusion by preventing interaction with its auxiliary receptor, proteolytic activation, and membrane fusion.^46^ Antibodies in this group are conformationally sensitive and also affected by mutations other than epitopes,^42^ such as several frequent NTD-located deletions (Δ69–70, Δ144, Δ157–158, and Δ211 and Δ242–244) in circulating VOCs (alpha and Omicron variant) (Fig. 2f).

Three RBM-directed antibodies (RBD-1/RBD-2/RBD-3 mAbs) recognize RBD in a manner nearly identical to that of ACE2 (Fig. 2g), meaning that the opened RBD conformation is required in the antibodies binding process. The differentiation lies in their respective epitope landscape, where RBD-1 mAbs (class II; such as CoVIC-259, EMD-24335) largely overlap with the RBM; RBD-2 mAbs (class III; such as CoVIC-252, CoVIC-010, CoVIC-140, and CoVIC-002) shift from the center of the ACE2 binding site toward the “Peak” of the RBM (consisted by residues F486, S477, T478, and E484); and RBD-3 mAbs (class IV; such as CoVIC-080, and EMD-24346) bind down from the center of ACE2 binding site toward the RBM “Mesa” (consisted by residues N501). These three types of antibodies block RBD-ACE2 interactions by both steric hindrance and direct competition for interface residues. Two classes of RBD mAbs (RBD-4 and RBD-5) can bind to the outer face of the open or closed RBD without steric hindrance (Fig. 2h), in which RBD-4 mAbs (class V; such as CoVIC-094, and EMD-24350) bind toward the outer edge of the RBM and most of them can block ACE2, whereas the epitope of RBD-5 nAbs (class VI; such as CoVIC-134, and EMD-24384) are away from the RBM and have weakly blocking to ACE2 binding.^42^ The remaining two groups of RBD mAbs (RBD-6 and RBD-7) recognize the inner face of the RBD, and two open RBDs are required in its targeting process (Fig. 2i). The main difference between RBD-6 (class VII; such as CoVIC-250, and CoVIC-028) and RBD-7 (class VIII; such as CoVIC-063, CR3022,^47^ and CoVIC-021) is their competition variance with RBD-2 (especially RBD-2a) antibodies, and the latter is superior to the former.^42^ All RBD-6 and partial RBD-7 (RBD-7a) antibodies are ACE2 blocking, and the rest of RBD-7 (including RBD-7b [such as CR3022] and RBD-7c [such as CoVIC-21]) are not ACE2 blocking.^42^

Only a few S2 mAbs (class IX) including have been reported to date, such as CC40.8,^48^ and 1A9,^49^ both of which belong to the cross-reactive antibody. Unlike 1A9, CC40.8 can interact with S2 subunit and neutralize SARS-CoV-2, while 1A9 only interacts with S2 subunit. In addition to the monoclonal antibodies mentioned above, polyclonal antibodies (pAbs) and antibody cocktails have also been proposed to combat the constantly emerging mutant strains.^28,50^

There are currently 137 COVID-19 vaccines in clinical development, of which recombinant protein subunit vaccines account for 35% (https://www.who.int/publications/m/item/draft-landscape-of-covid-19-candidate-vaccines). In the process of vaccine development, a series of obstacles have been encountered. For example, full-length recombinant S glycoprotein is difficult to express and has poor stability; recombinant RBD vaccines recognize fewer neutralizing epitopes compared with full-length S protein vaccines and are prone to antigenic drift;^16^ and the extensive glycosylation of S glycoprotein makes it easy for the coronavirus to escape host immune attack.^51,52^ Employing the methods of structural determination, some groups have found that the more stable closed prefusion S glycoprotein can be obtained by introducing single point mutations and disulfide bridges.^53,54^ Furthermore, several key residues associated with glycosylation have been identified, including N165, N234, and T323 of the S protein.^55–57^ All of this structural information provides an important theoretical basis for further vaccine optimization.

In addition to the S protein, other proteins involved in the process of virus-membrane fusion can also be targeted. ACE2 is a host receptor of S protein and is highly expressed in many tissues (including nasal mucosal, bronchus, lung, heart, kidney, and intestinal tissue)^58^ and regulated by the amino-acid transporter B^0^AT1.^59^ Some inhibitors (such as DX600,^60^ MLN4760,^61^ and NAAE^62^) targeting ACE2 effectively inhibit entry of the virus. Host proteases, such as cathepsins,^63^ furin,^28^ TMPRSS2,^64^ and trypsin are another set of potential targets. The S protein is cleaved by host proteases at the S1/S2 and S2’ sites, activating the S protein and allowing it to mediate membrane fusion.^65,66^ Inhibitors of furin protease include decanoyl-RVKR-chloromethylketone (CMK)^67^ and a-1 antitrypsin Portland (a1-PDX).^68^ There are other known host receptors of S protein, such as glucose-regulated protein 78 (GRP78)^69^ and CD147,^70^ which could also be targeted.

### Effect of S protein mutations on SARS-CoV-2 immune evasion

To date, there are five globally recognized SARS-CoV-2 variants of concern (VOCs; Alpha, Beta, Gamma, Delta, and Omicron) and several variants of interest (VOIs; Epsilon, Zeta, Eta, Lota, Theta, Kappa, Lambda, and Mu) are emerged under the pressure of natural selection of the human immune system. Among these variants, the most diverse regions in virus genomic are distributed in NTD and RBD of S protein (Table 1). These mutations have direct implications on virus virulence (infectivity or reinfection, transmissibility, disease severity, neutralization resistance to antibodies elicited by infection or vaccination), and thus potentially resulted in more obstacles to the precaution, diagnose, and therapy of COVID-19. The common characteristics of these variants and factors associated with virus virulence variance are discussed below.

**Table 1:** Summary of characteristic of variants of interest (VOIs) and concern (VOCs)

Variants	Mutations in the S protein				Potential drugs^a^
	NTD	RBD	S1-CTD	S2 domain	
Alpha (B.1.1.7)	Δ69–70, ΔY144,	N501Y,	A570D, P681H	T716I, S982A, D1118H	Most of RBD-directed mAbs
Beta (B.1.351)	L18F, D80A, D215G, R246I,	K417N, E484K, N501Y	D614G,	A701V	Some mAbs (such as C135, COVA1–16, S309, REGN10987, AZD1061, AZD7442, mAb222, Casirivimab and imdevimab),^360,361^ and PF-07321332^362^
Gamma (P.1)	L18F, T20N, P26S, D138Y, R190S,	K417T, E484K, N501Y,	D614G, H655Y,	T1027I,	Some mAbs (such as S309, mAb222, AZD7442, AZD1061, and REGN10987, Casirivimab and imdevimab)^360,363^
Delta (B.1.617.2)	T19R, G142D, Δ156–157, R158G,	L452R, T478K,	D614G, P681R,	D950N	Some mAbs (such as Sotrovimab, BRII-198, BRII-196, Bamlanivimab, Etesevimab, Casirivimab, and imdevimab)^364,365^ and Molnupiravir^366^
Epsilon (B.1.427/9)	S13I, W152C	L452R	D614G	/	Nucleoside analog Sangivamycin^367^
Zeta (P.2)	L18F, T20N, P26S, F157L,	E484K,	D614G,	S929I, V1176F	Nucleoside analog Sangivamycin; and PF-07321332^362^
Eta (B.1.525)	Q52R, A67V, Δ69–70, Δ144,	E484K,	D614G, D677H	F888L	Some mAbs, such as Casirivimab and imdevimab
Lota (B.1.526)	L5F, T95I, D253G,	S477N, E484K,	D614G	A701V	Some mAbs, such as Casirivimab and imdevimab
Kappa (B.1.617.1)	G142D, E154K	L452R, E484Q,	D614G, P681R,	Q1071H	Some mAbs, such as Casirivimab and imdevimab; Molnupiravir^366^
Lambda (C.47)	G75V, T76I, Δ246–252,	L452Q, F490S,	D614G	T859N	Some mAbs (such as Casirivimab and imdevimab) and PF-07321332
Mu (B.1.621)	T95I, Y144S, Y145N,	R346K, E484K, N501Y,	D614G, P681H	D950N	Molnupiravir^366^
Omicron (B.1.1.529)	A67V, Δ69–70, T95I, G142D, Δ143–145, Δ211, Y145D, L212I, ins214EPE,	G339D, S371L, S373P, S375F, K417N, N440K, G446S, S477N, T478K, E484A, Q493R, G496S, Q498R, N501Y, Y505H,	T547K, D614G, H655Y, N679K, P681H,	N764K, D796Y, N856K, Q954H, N969K, L981F	(1) Some mAbs: VIR-7831, VIR-7832, DXP-604 and BRII-198 (2) Ribonucleoside analogs that target to Nsp12 (3) Protease inhibitors that target to Nsp3, Nsp5 and other protease participated in virus infection

^a^Vaccines in this table are not listed, and vaccines efficacy and effectiveness against variants can be obtained at https://www.who.int/publications/m/item/draft-landscape-of-covid-19-candidate-vaccines. Moreover, booster vaccination is still the best strategy to prevent SARS-CoV-2 variants at present

S13I, L18F, 69–70del, 141–143del, 144del, W152C R246I/M, K417N/T/M, L452R, N440K, S477G/N/R, E484K/A/Q/P, Q493K/R, N501Y, H655Y mutations are associated with antibody evasion

S477G/N/R, N501Y, D614G mutations increase the binding affinity of S protein to ACE2

L452R, P681H/R mutation increase virus transmissibility

69–70del, P681H/R increase virus infectivity

69–70del, K417N mutation is related to the conformational change of S protein

Alpha variant has increased transmissibility, and disease severity than pre-existing variants. This variant has seven missense mutations and three deleted residues in the S protein (Table 1). Among these mutations, N501Y mutation is shared by several VOCs (Alpha, Beta, Gamma, and Omicron) and VOIs (Theta and Mu), and this mutation increase the binding affinity of S protein and ACE2 receptor by adding an additional hydrogen bond between them, and consequently raising the efficiency of viral infection and transmission.^71^ D614G mutation increases the ability of RBD to shift to the up position, and is more conducive to the transmission and infection of the virus.^72^ P681H mutation is adjacent to the furin cleavage site and could potentially have an effect on S1/S2 cleavage and therefore on cell entry and infectivity.^73^ Three deleted residues in NTD (69–70, Y144) are recurrent mutation sites observed in VOCs and VOIs, and likely responsible for the resistance of these variants to neutralization by NTD-specific antibodies.^74^ Fortunately, the Alpha variant remains sensitive to neutralization by currently potential antibodies, but its level is moderately reduced.^75^

The second dominant VOCs is Beta variant, and ~77% of mutations in its genomic located in the S protein include seven missense mutations and three deleted residues (Table 1).^75^ Multiple studies have shown that Beta variant displayed neutralization resistance to most NTD-, and RBM-specific mAbs, and this resistance is mainly ascribed to three mutations within RBD (K417N, E484K, and N501Y).^76^ N501Y probably does not impair neutralization on its own but rather in combination with K417N and E484K, both mutations play contributions to the enhanced binding affinity of S protein with ACE2 and increased immune evasion capability of variants.^75^ Furthermore, the combination of RBD and NTD mutations in the S protein would affect the neutralization more significantly.

The Gamma variant is the third VOC. This variant contains eleven S protein mutations, including five mutations within NTD (Table 1), three mutations in RBD that resemble to RBD mutations in the Beta variant (K417N, E484K, and N501Y), two mutations near the furin cleavage site (D614G and H655Y), and one mutation in S2 (T1027I). Gamma variant has significant resistance to the neutralizing antibodies from convalescent and vaccinee sera, but the degree of resistance is not as severe as against Beta and only slightly weakened compared to that of Alpha.^77^ The reason for the differences in neutralization resistance of Beta and Gamma presumably reflects the difference in the mutations accumulated outside the RBD.^77^

The presently dominated VOCs in the global is still Delta variant, which may be over 60% more transmissible than the Alpha variant.^77^ This variant is characterized by numbers of spike mutations, including five NTD mutations (Table 1), three RBD mutations (L452R, T478K, and E484K), and two mutations near the furin cleavage site (D614G and P681R), and one S2 mutation (D950N). L452R mutation has been proved to be associated with increased infectivity, higher transmission, and immune evasion of SARS-CoV-2 variants.^78^ T478K mutation was previously selected in vitro and shown to exhibit reduced neutralization by monoclonal antibodies and human convalescent sera.^79^ The significantly increased immune escape capacity to neutralize antibodies of this variant may be related to the RBD mutations and their combination with NTD mutations.

The recently emerged Omicron variant, with higher transmissibility, infection/reinfection rate, and enhanced ability of immune escape, are attracting more and more attention.^80^ This variant has more than 50 mutations, and most of the mutations (at least 32) were located within S protein, with half of these mutations are located in the RBD (Table 1) and 10 of them are concentrated in the RBM.^81,82^ Although some mutations (such as Δ69–70, T95I, G142D, Δ143–145, K417N, T478K, N501Y, H655Y, N679K, and P681H) that overlapped with those in other VoCs are have been investigated to participate in viral transmissibility, binding affinity, and immune escape; while the role of the most of the remaining mutations is still not known. Therefore, it is urgent to study the effect of these mutations on virus virulence, antigenicity, and epidemiology, as well as neutralization activity to antibodies. In addition to the mutations in S protein, only one mutation in each of these genes (Nsp14 and Nsp5) are found, so inhibitors that are targeted these proteins may be useful for Omicron variant.^83^ Some approved or potential representative drugs targeting SARS-CoV-2 proteins were listed in Table 2.

Some essential mutations mentioned above (L452R, E484K/Q, N501Y, D614G, and P681H/R) also presented with some significant VOI variants, such as Epsilon, Eta, Lota, Kappa, Zeta, and Theta. Their respective mutation information in S protein is listed in Table 1.

### N protein

The multifunctional nucleocapsid (N) protein not only participates in viral replication and assembly, but also interferes with the interferon pathway of the host.^84,85^ Compared with the more researched drug targets (S protein, 3CLpro, and PLpro), the N protein has several advantages: it has higher sequence conservation, is less prone to mutation, and induces a stronger protective immune response in the host. Therefore, N protein is a great potential target for diagnosis.

**Table 2:** Overview of targets and potential drugs of SARS-CoV-2

Drug targets	Approved/potential COVID-19 drugs
S	Ivermectin,^36^ Arbidol,^38^ Lipopeptide EK1 and EK1C4,^41^ SSA09E2,^368^ Griffithsin,^369^ Nidazolamide and Tizolazolide,^370^ Heparin,^371^ Withaferin A,^372^ Tetracycline,^373^ Monoclonal antibodies targeted to S protein
N	Rapamycin,^93^ PJ34^94^
E	Hexamethylene amiloride (HMA) and Amantadine (AMT),^98^ Hm-methyleneamelory,^374^
Nsp1	Glycyrrhizic acid, Lobaric acid, Garcinolic acid and Tirilazad^139^
Nsp2	Nigellidine,^146^
Nsp3	VIR251,^173^ GRL-0617,^151^ YM155,^175^ Ribavirin,^375^ GRL-0667 and Mycophenolic acid^376^
Nsp4	Eribulin and Suvorexant^184^
Nsp5	PF-07321332 (Nirmatrelvir), S-217622, PF-07304814, PBI-0451, Enanta (EDP-235), N3,^190^ Calpain inhibitors II, Calpain inhibitors XII, GC-376, Baicalin, Baicalein, and Boceprevir,^377^ α-ketoamide 13b,^192^ Andrographolide,^378^ Hispidin and Lepidine E,^379^ Talampicillin, and Lurasidone,^380^ Vitamin B12 and Folic acid,^381^ Pitavastatin; Leupeptin; hemisulfate; pepstatin A; Birinapant; Lypression; octreotide; Nicotinamide, Famotidine; Telbivudine, and Telcoplanin,^382^ Poziotinib; Fostamatinib; Ziprasidone; Telcagepant; Talampicillin; Hypericin; Cyanidin-3-glucoside; Glabridin; α-ketoamide 11r; Cannabisin A; Isoacteoside; Raltegravir, Dolutegravir, Bictegravir, Paritaprevir, Darunavir, and Oseltamivir,^383^ Nelfinavir and Nelfinavir Mesylate,^384^ Ritonavir and Lopinavir,^385^ Elbasvir; Favipiravir, Hydroxychloroquine and Chloroquine,^386^ PF-07321332,^194^
Nsp6	Dextromethorphan, and Haloperidol,^184^ Sucrose (ZINC000004217475),^387^
Nsp9	Uracil-analog FR6,^216^ imidazolium salts,^218^ Teicoplanin, and Azithromycin^219^
Nsp12	Ribavirin, Penciclovir, Tenofovir, Sofosbuvir, and Galidesivir,^239^ Favipiravir,^236^ EIDD-1931,^388^ Molnupiravir (EIDD-2801),^238^ Remdesivir,^234^ Surami,^247^ Zn (II),^389^; Vitamin B12,^390^ YAK, Setrobuvir and IDX-184,^391^ Cefuroxime,^392^ ODBG-P-RVn,^393^ GS-621763,^394^
Nsp13	Bananins,^395^ Lymecycline, Cefsulodine, Rolitertracycline, Itraconazole, Saquinavir, Dabigatran, and Canrenoic acid,^396^ Rolitetracycline,^375^
Nsp14	S-adenosyl-homocysteine (SAH) and Sinefungin (SFG),^308^ Aurintricarboxylic acid (ATA),^283^ Disulfiram and Ebselen,^281^
Nsp15	Tipiracil,^289^ NSC95397,^397^ Benzopurpurin B, C-473872, and Congo red,^398^
Nsp16	S-adenosyl-homocysteine (SAH) and Sinefungin (SFG),^308^ Aurintricarboxylic acid (ATA),^283^ Withanolide (WTL), and Dolutegravir,^399^ Bictegravir,^400^
ACE2	DX600,^60^ MLN4760,^61^ NAAE,^62^ Telmisartan,^401^ Chloroquine and Hydroxychloroquine,^402^ Ibuprofen,^403^ ACEI,^404^ ACE inhibitors, Soluble recombinant human angiotensin-converting enzyme 2 (rhACE2)^405^
Cathepsin L	Calpain inhibitors, E64d and Inhibitor SID26681509,^406^ MDL28170,^407^
Furin	Furin inhibitors, Furine convertase inhibitors, Decanoyl-RVKR-chloromethylketone (CMK) and Naphthofluorescein,^408^ α1-PDX,^409^ Peptidyl-chloromethylketone, 2,5-dideoxystreptamine-derived inhibitor^374^
TMPRSS2	Nafamostat and Nafamostat Mesylate,^410^ Camostat and Camostat Mesylate,^411^ Bromhexine hydrochloride,^412^ Benzoselenoxanthene analogues,^413^ Cbz (Carboxybenzyl)-Phosphono-Lys (OPh)2,^414^ Rubitecan, Loprazolam; Compounds ZINC000015988935, and ZIN C000103558522,^415^ Estradiol,^416^ Enzatulamide^417^
CD147	Meplazumab,^418^ Azihromycin,^419^ Doxycycline^420^
DPP4	Sitagliptin^421^
Other targets	Lianhua Qingwen,^20^ Interferon,^422^ Vitamin C,^423^ Tocilizumab^424^

**Fig. 3:**
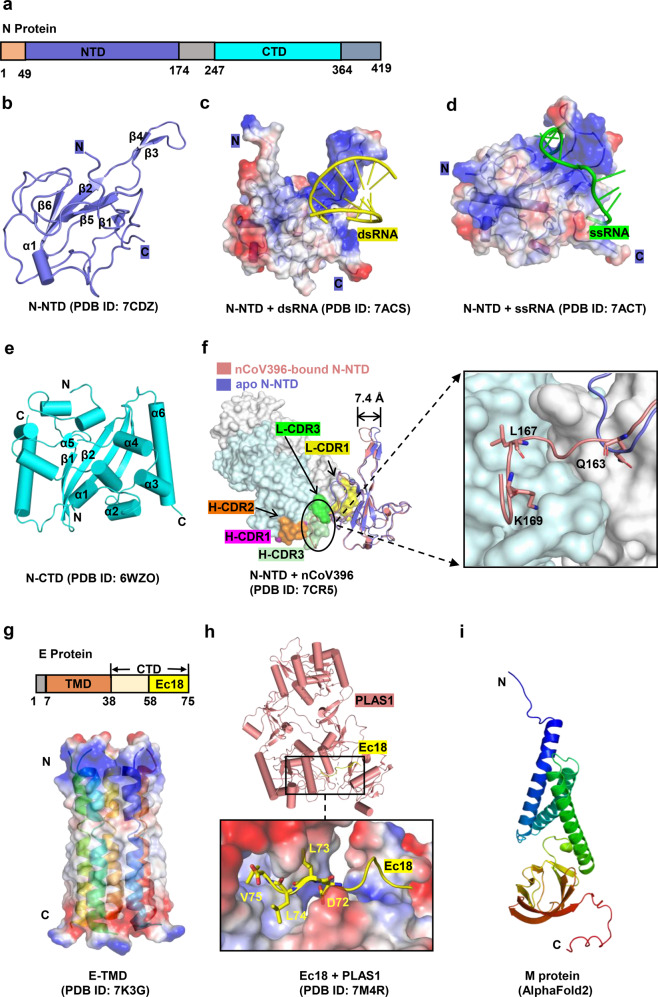
Structures of SARS-CoV-2 Nucleocapsid, Envelope, and Membrane proteins. **a** Schematic representation of N protein organization. NTD N-terminal domain, CTD C-terminal domain. **b** Overall structures of N-NTD. **c, d** Overall structures of N-NTD in complex with ssRNA and dsRNA, respectively. The N-NTD is illustrated with electrostatic surface. **e** Overall structure of N-CTD. **f** Conformational changes between apo and nCoV396-bound N-NTD. The monoclonal antibody nCoV396 is illustrated with surface. The key residues of interaction between nCoV396 and N-NTD are shown on the right as sticks. **g** Overall structure of SARS-CoV-2 E protein shown in surface and cartoon. TMD transmembrane domain, CTD C-terminal domain, Ec18 residues 58–75 of E-CTD. **h** Structure of the C-terminus of E protein (Ec18) in complex with human cell junction protein PLAS1. The Ec18 is shown in yellow. **i** Predicted structure of M protein using alphafold 2

The SARS-CoV-2 N protein shares 90% homology with the SARS-CoV N protein.^86^ SARS-CoV-2 N comprises 419 amino acids, which are divided into the N-terminal domain (residues 49–174), the C-terminal domain (CTD, residues 247–364), and a flexible linker (residues 175–246) (Fig. 3a). The linker is rich in serine/arginine (SR, residues 176–206) and leucine/glutamine (LQ, residues 210–246), and SR regions play an essential role in primary phosphorylation.^87^ The structure of the full-length N protein has not yet been resolved, but specific structural features are known. The N-NTD folds into a right-hand shape, in which six antiparallel β-sheets (β4-β2-β3-β1-β5-β6) form the palm and a protruding positively charged β-hairpin located between β2 and β3 forms the basic finger^88^ (Fig. 3b). A typical characteristic of the N protein is that it binds to viral RNA and forms a ribonucleoprotein complex (RNP).^89^ In the N-RNA oligonucleotide complex (Fig. 3c, d), the negatively charged RNA binds to the positively charged canyon in the N protein. RNA interacts with several basic residues (R92, R107, and R149) and causes a significant conformational change in the basic finger.^85,90^ The CTD domain of the N protein is assembled into a cuboid composed of homologous dimers, and each protomer consists of six α-helices and two β-strands (Fig. 3e). The α-helices and β-strands of one protomer are embedded in the cavity of the other protomer, forming four-stranded antiparallel β-sheets. Pairs of hydrogen bonds and hydrophobic interactions between the two adjacent β-sheets cooperatively stabilize the structure of the dimer.

The N protein is one of the most abundant structural proteins in virus-infected cells,^91^ and can be used as an immunodominant antigen to induce a protective immune response. Monoclonal antibody nCoV396 isolated from the blood of convalescent COVID-19 patients effectively interacts with the NTD of the N protein (1.02 nM).^92^ Its heavy chain and light chain contain multiple CDRs. such as, L-CDR1 (residues 23–32), L-CDR2 (residues 51–54), L-CDR3 (residues 94 to 100), H-CDR1 (residues 26–33), H-CDR2 (residues 51–57), and H-CDR3 (residues 99–108). These CDRs form hydrogen bonds and hydrophobic interactions with three residues of the N-NTD (Q163, L167, and K169) to stabilize the protein complexes. Notably, these three residues are relatively conserved in the highly pathogenic β-coronavirus N protein. nCoV396 causes a large allosteric regulation of the N-NTD, such as outward movement of the basic finger by 7.4 Å and increased unfolding of the carbon end tail (residues 159–172)^92^ (Fig. 3f). These interactions synergistically benefit the antibody in neutralizing the antigenicity of the N protein. nCoV396 also has a high affinity with the N protein of SARS-CoV and MERS-CoV (7.4 nM).^92^ In the future, this antibody could be modified in a site-directed manner based on specific structural information to allow the production of more efficient and broader spectrum monoclonal antibodies. In addition to antibodies, some small molecules (such as rapamycin^93^ and PJ34^94^) that interfere with the RNA binding of N-NTD and dimerization of N-CTD have also been recommended as inhibitors of the N protein.

### E protein

The envelope (E) protein forms the cation-selective channel in the endoplasmic reticulum-Golgi intermediate compartment (ERGIC).^95^ The E protein not only assists in viral assembly, budding, and virulence^96^ but also induces the host NACHT, LRR and PYD Domain-Containing protein 3 (NLRP3) inflammasome.^97^ As a critical multifunctional structural protein, the E protein has attracted increasing attention from researchers.

SARS-CoV-2 encodes a transmembrane E protein composed of 75 amino acids, including the N-terminal ectodomain (residues 1 to 7), transmembrane domain (TMD) (residues 8–38), and C-terminal domain (residues 39–75), with the 18 amino acids at the C-terminus referred to as Ec18. The structure of TMD is a pentameric ion channel (PDB ID 7K3G) formed by five α-helices^98^ (Fig. 3g). The stability of the adjacent α-helix and helical bundle is mediated by π–π stacking (Phe23 and Phe26), Van der Waals packing (among the Val29-Leu31-Ile33 triad), and extensive hydrophobic interactions (among the abundant hydrophobic residues in the pore of the channel).^98^ The guanidinium groups of two ion channel drugs, hexamethylene amiloride (HMA) and amantadine (AMT), interact with the polar amino acids at the entrance of the ion channel and occupy the amino-terminal lumen of the channel, blocking ion channel activity of the E protein.^98,99^ The postsynaptic density-95 (PSD-95), discs-large, zona occludens 1 (ZO-1) (PDZ)-binding motif (PBM:^72^DLLV^75^) at the C-terminus of the E protein recognizes the PDZ domain of the human cell junction protein PALS1 and subsequently breaks the apical cell polarity complex formed by PALS1, Crumbs, and Pals1-associated tight junction protein (PATJ).^100–102^ This causes looseness and leakage of the lung epithelial junctions and facilitates viral spread and proliferation.^103,104^ In the PLAS1/Ec18 complex (Fig. 3h) (PDB ID 7M4R), two PALS1 proteins interact with one Ec18, and the DLLV motif of Ec18 occupies the hydrophobic pocket formed by the PDZ domain and SH3 domain of PLAS1.^103^ This pocket is also the binding site of the ERLI motif at the C-terminus of Crumbs (CRB-CT) in PLAS1 (PDB ID 4WSI).^100,105^ Therefore, similar peptide inhibitors derived from Ec18 can be designed to inhibit the interaction between the E protein and PLAS1.^103^ In addition to the two inhibitors mentioned above, several other drugs target the E protein, such as Bacillus Calmette-Guerin (BCG) vaccination;^106^ this is an alternative strategy for the treatment of COVID-19 that elicits specific host immunity targeting the SARS-CoV-2 E protein.^107,108^ A possible reason for this response is that a fragment of the E protein (residues 17–29) has a high sequence identity with tuberculin-like proteins.^107^

### Predicted structure of M protein

Membrane glycoprotein (M), the most abundant protein in coronaviruses,^109^ is the main component of the viral envelope and maintains the virion’s size and shape.^110^ Moreover, M protein is involved in the processing, modification, and trafficking of multiple viral proteins,^111^ as well as the assembly and release of virus particles.^112^ M protein also interferes with the host immune response through interferon antagonism.^113^ Therefore, it is a promising target for the treatment of COVID-19.

The SARS-CoV-2 M protein shares 90.5% sequence identity with SARS-CoV M protein.^114^ However, M protein has been proven difficult to be expressed and purified, hindering progress in resolving the crystal structure and leaving only a predicted structure available.^114^ The SARS-CoV-2 M protein consists of 222 amino acids, including a short N-terminus (residues 1–19), a triple-transmembrane domain (TM, residues 20–100), and a longer C-terminal cytoplasmic domain (residues 101–222). The predicted structure of the M protein is similar to the prokaryotic SemiSWEET sugar transport protein,^115^ with both containing three transmembrane helix bundles (Fig. 3i). The M protein contains several conserved motifs, which are not only responsible for the homodimerization and translocation of the M protein, but also participate in the interaction between the M protein and other viral proteins.^113,116^ For example, “aromatic-X-X-aromatic” regions (such as WLLW in TM2 [residues 50–70]) are closely related to the dimerization of the M protein,^117^ and the di-leucine motif located at the C-terminal tail of the M protein interacts with the N protein.^118^ Research into these conserved motifs is expected to contribute to the development of drugs targeting this protein.

The SARS-CoV M protein has strong immunogenicity and stimulates the host humoral response to produce neutralizing antibodies.^119^ Multiple cytotoxic T-lymphocyte (CTL) related epitopes of M protein have been identified by human leukocyte antigen (HLA) molecule (HLA-A^*^0201), such as Mn2 (residues 88–96) and Md3 (residues 60–69), located in the TM region.^120^ They all stimulate the host to produce a specific CD8^+^ T cell immune response.^120^ In the HLA-A^*^0201-Mn2 (PDB ID 3I6G) or HLA-A^*^0201-MD3 (PDB ID 3I6K) complex, several anchor residues (such as leucine, valine, methionine, and serine) in Mn2/Md3 assist in tightly binding peptides to the HLA pocket.^120^ In the future, the same approach used to identify the CTL-related epitopes of the SARS-CoV M protein can also be applied to screen the epitopes of the SARS-CoV-2 M protein, allowing for the design of peptide inhibitors or vaccines against this protein. Recently, some groups have used molecular dynamics (MD) simulations and other strategies to identify several potential drugs (such as remdesivir) with high affinity with the M protein,^121^ but further studies are needed to confirm these interactions.

## Nonstructural proteins of SRAS-CoV-2

Currently, the drugs and vaccines used to treat or prevent COVID-19 are predominantly targeted to the S protein, but the rapid mutation in this region can easily lead to drug resistance. Therefore, it is necessary to develop drugs that target other proteins. SARS-CoV-2 encodes sixteen nonstructural proteins (Nsp1–Nsp16) that form the replication and transcription complex (RTC). The transmembrane proteins Nsp3, Nsp4, and Nsp6 hijack and rearrange the host endoplasmic reticulum membrane, then induce the formation of double-membrane vesicles (DMVs).^122,123^ As an organelle-like structure, the DMV is not only conducive to viral replication but also assists in the evasion of the host’s innate immune response. Among these nonstructural proteins, the structure of the three transmembrane proteins is the most complicated, all consisting of multiple transmembrane domains and luminal domains.^124^ Nsp11 is a short peptide composed of 7 amino acids,^125^ and the structures of Nsp4 and Nsp6 remain unresolved; we, therefore, focus here on the other thirteen Nsps, and the predicted structures of Nsp4 and Nsp6 are also referred to.

### Nsp1 protein

Nsp1 is an essential virulence factor of coronaviruses and is closely associated with the viral infection cycle and host translation regulation.^126^ It specifically binds to the host 40 S ribosomes and promotes endonucleolytic cleavage of host mRNA, thus hijacking the translation of multiple host genes. These genes include type-I interferon,^127–129^ allowing the virus to evade the host’s innate immune defense.^130,131^ Therefore, recombinant viruses with mutated Nsp1 can be used to design live attenuated vaccines.^132,133^

**Fig. 4:**
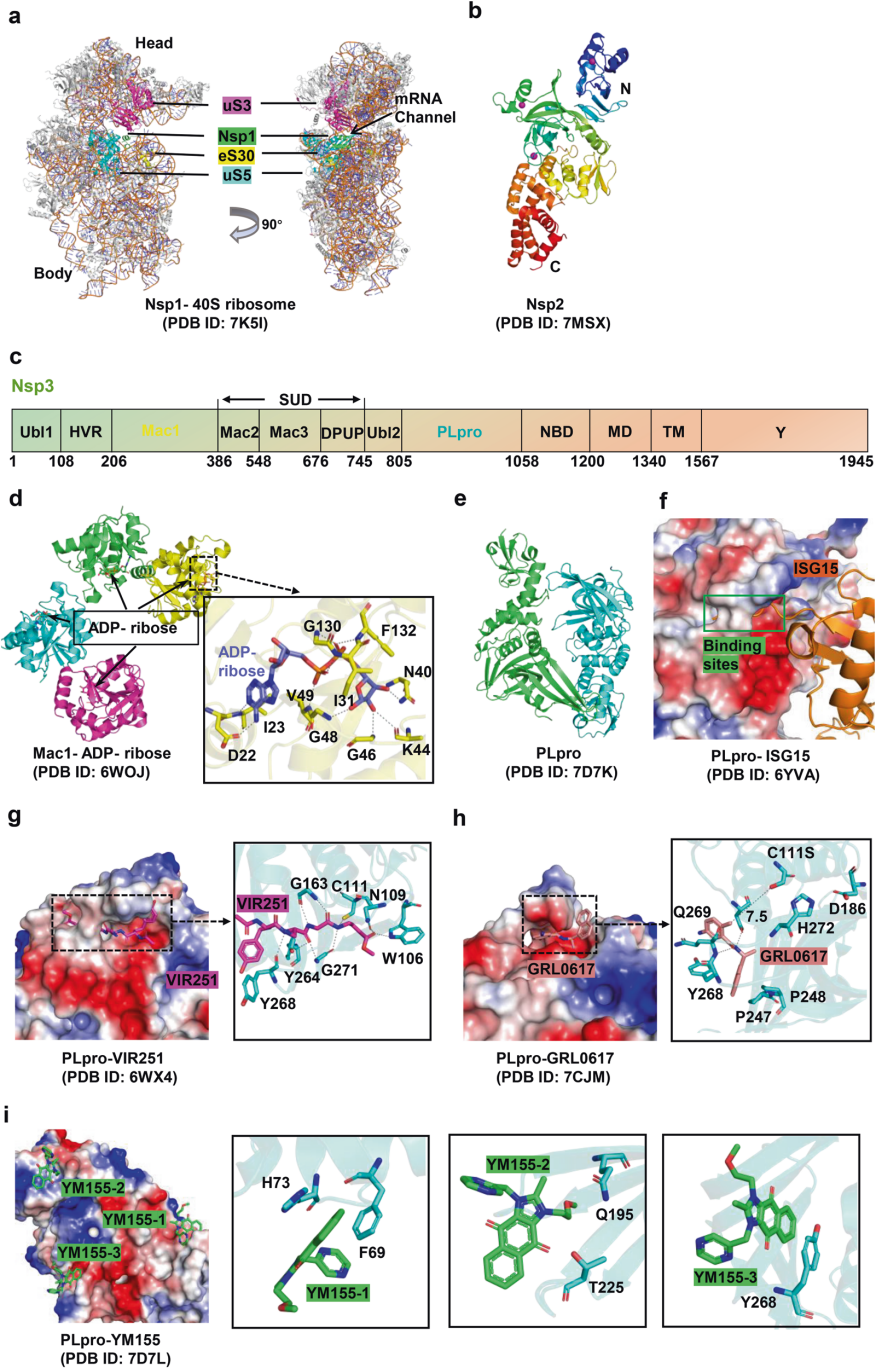
Structures of Nsp1 /Nsp2 /Nsp3 and their inhibitors. **a** Structure of Nsp1 in complex with 40 S ribosome. Nsp1 binds at the mRNA channel in the cleft between the head and body of the 40 S ribosome. **b** Overall structure of Nsp2 protein. **c** Schematic representation of Nsp3 protein’s domain. **d** Structures of Mac1 domain in complex with ADP ribose. **e, f** Structure of apo PLpro and its complex with interferon-stimulated gene 15 protein (ISG15), respectively. **g**–**i** Structures of PLpro in complex with different inhibitors (VIR251, GRL-0617, and YM155). The PLpro is illustrated with electrostatic surface

SARS-CoV-2 Nsp1 consists of 180 amino acids and shares high structural homology but low sequence identity with other Nsp1 proteins of β-coronaviruses (with the exception of SARS-CoV). In the N-terminal domain (NTD, residues 10–127) (PDB ID 7K7P), seven antiparallel β-strands assemble a closed β-barrel. An α-helix is located at the opening of the barrel as a cap, and two 3_10_ helices are fixed on one side of the barrel.^134^ The C-terminal domain (CTD, residues 148–180) specifically interacts with the 40 S ribosomal subunit.^135^ In the Nsp1-CTD/40 S ribosome complex structure (Fig. 4a), Nsp1-CTD is embedded into the mRNA entry channel in the 40 S ribosome subunit.^135–137^ The stability of the complex depends on the electrostatic and hydrophobic interactions between Nsp1-CTD and 40 S ribosome protein subunits (uS3, uS5, and eS30), as well as the 18 S rRNA. The roles of Nsp1-NTD in the modulation of translation have been discussed; it not only stabilizes the binding of Nsp1-CTD to 40 S ribosome^138^ but also specifically interacts with stem-loop 1 (SL1) of the SARS-CoV-2 mRNA 5′UTR,^136,139^ meaning that Nsp1 only inhibits translation of host genes. Some inhibitors (such as glycyrrhizic acid, lobaric acid, garcinolic acid, and tirilazad) that destroy the interaction between Nsp1 and SL1 are expected to be effective in COVID-19 treatment.^139^

### Nsp2 protein

Nsp2 is an endosome-associated protein with unknown specific functions. It interacts with multiple host proteins (such as Prohibitin 1[PHB1], PHB2, and actin-nucleation-promoting WASH protein)^140–142^ and participates in biological processes such as viral replication, host immune regulation, mitochondrial biogenesis, and endosomal transport.^143,144^ Nsp2 is therefore a promising novel target for the treatment of COVID-19.

SARS-CoV-2 Nsp2 comprises 638 amino acids, and its full-length structure has been obtained using cryo-electron microscopy in conjunction with AlphaFold2 structural prediction^141^ (Fig. 4b). The N-terminus of Nsp2 (residues 1 to 276) consists of ten α-helices, fourteen β-strands, and three classic zinc-finger (ZnF) structures: C2H2 ZnF, C4 ZnF, and C2HC ZnF.^145^ The residual structure of Nsp2 (residues 277–635) is relatively simple. Its carbon terminus consists of only 14 β-strands, some of which form highly disordered loops, and one 3_10_ helix. The middle region contains three β-strands and nine α-helices. The Nsp2 protein binds nucleic acids nonspecifically,^141,145^ with the binding site being either ZnFs^141^ or the positively charged region on the surface of Nsp2;^145^ the specific identity of the binding site is controversial.

There are currently no verified inhibitors of Nsp2. Some potential Nsp2 inhibitors are derived from molecular docking. For example, nigellidine,^146^ an indazole-alkaloid, may bind to the entry pocket of Nsp2. It forms a hydrogen bond with Cys240 of Nsp2 (3.26 Å), and thus occupies Nsp2 entry channel formed by several residues (Leu169, Val126, Trp243, Ala127, Cys132, Thr256, Gly257, Tyr242, and Val157).^146^ Notably, the increased transmissibility and pathogenicity of some SARS-CoV-2 variants are closely related to Nsp2 mutations, such as T85I of Nsp2 in the B.1.526/B.1.427/B.1.429 variant.^147–149^ The presumed explanation is that mutations interfere with the interaction between Nsp2 and host proteins,^141^ ultimately affecting virulence. The immunogenicity of Nsp2 can be applied to the development of inactive or live attenuated virus vaccine.

### Nsp3 protein

Nsp3 (also called papain-like protease, PLpro) is the largest membrane-associated cysteine protease produced by coronaviruses; it recognizes the tetrapeptide LXGG-motif and hydrolyzes viral polyprotein pp1a to generate three nonstructural proteins (Nsp1, Nsp2, and Nsp3).^150^ A versatile protein, PLpro not only hydrolyzes ubiquitin and ubiquitin-like interferon-stimulated gene 15 protein (ISG15) but is also involved in post-translational modification of host proteins (de-ubiquitination and de-ISGylation). This role allows it to interfere with the host immune response, especially the interferon and NF-κB pathways.^151–156^ In summary, Nsp3 plays a critical role in viral reproduction and suppressing the host immune response, and is, therefore, an attractive drug target for the treatment of COVID-19.

SARS-CoV-2 Nsp3 consists of 10 domains, including the ubiquitin-like domain 1 (Ubl1, residues 1–108), hypervariable region (HVR, residues 109–206), macrodomain I (Mac1 or X, residues 207–386), “SARS-unique domain” (SUD; composed of three subdomains: macrodomain II [Mac2, residues 387–548], macrodomain III [Mac3, residues 549–676], and domain preceding Ubl2 and PL2pro [DPUP, residues 677–745]), ubiquitin-like domain 2 (Ubl2, residues 746–805), papain-like protease (PLpro, residues 806–1058), nucleic acid-binding domain (NBD, residues 1059–1200), marker domain (MD, residues 1201–1340), transmembrane regions (TM, residues 1341–1567), and the Y domain (residues 1568–1945)^157–159^ (Fig. 4c). The Ubl1, Mac1, and PLpro domains are discussed below in detail.

The core region of the Ubl1 domain (residues 18–109) folds into a canonical ubiquitin-like shape (β1-α1-β2-α2-3_10_-β3-β4) (PDB ID 7KAG), which resembles the structure of human ubiquitin (Ub) and two ubiquitin-like domains in human and mouse interferon-stimulated gene 15 (hISG15 and mISG15).^157^ Ubiquitin-like modules are often involved in protein–protein interactions to regulate various biological processes.^91,160^ Distinct from Ubl2, the function of which is uncertain, Ubl1 specifically binds ssRNA with AUA patterns and interacts with the N protein.^161^ The latter interaction is essential for viral replication and pathogenicity.^91^ The interface regions of the Ubl1-N complex involve acidic residues of the Ubl1 helix α2 and the SR-rich region of the N protein.^91,162,163^

In the processes of viral infection, the Mac1 domain counteracts host-mediated antiviral adenosine diphosphate-ribosylation signaling via its ADP-ribosyl hydrolase activity.^164,165^ Accordingly, catalytic null mutations of the Mac1 domain render viruses nonpathogenic,^164–166^ and the Mac1 domain is a promising drug target for disrupting the viral life cycle. The Mac1 domain adopts a conserved three-layered α/β/α sandwich fold, in which there is a central seven-stranded β-sheet (β1-β2-β7-β6-β3-β5-β4), and six α-helices are located on the outside.^157,167^ The Mac1 domain contains four substrate-binding pockets, namely the adenine-binding, distal ribose-binding, diphosphate-binding, and proximal ribose-binding sites. In the Mac1 domain-substrate (ADP-ribose [ADPr]) complex (Fig. 4d), ADPr is located in a cleft at the top of the central β-sheet.^167,168^ The adenine moiety is in a mostly hydrophobic environment, and its N6 and N1 atoms form hydrogen bonds with Asp22 and Ile23, respectively. The proximal ribose ring is stabilized in the pocket by several hydrophobic interactions with Phe132/Ile13, and hydrogen bonds with Gly46/Gly48/Asn40. The α-/β-phosphate group is located in a narrow channel formed by loops β3-α2 and β6-α5, and accepts hydrogen bonds from Val49 and Gly130/Phe132, respectively.^167,169^ The distal ribose ring only participates in water-mediated hydrogen bonds with Leu126, Ala154, and Asp157. Asp22 and Asn40 appear to fix the two ends of ADPr. PDD00017273 is the only well-characterized inhibitor of macrodomain-type (ADP-ribosyl) hydrolase.^164,170^ Some small fragments that bind to the Mac1 domain have been identified using a combination of computational and structural analysis. The screened inhibitors occupy different sites in the Mac1 domain; these include the four substrate-binding sites mentioned above and the oxyanion subsite (adjacent to the adenine subsite and formed by Phe156/Asp157).^164,171,172^

PLpro has proteolytic, deubiquitinating (DUB), and deISGylating activity.^173^ The overall structure resembles a cellular DUB protein from herpesvirus (Fig. 4e), which folds into a right-handed shape composed of five domains: a palm domain, a thumb domain, a finger domain, the N-terminal ubiquitin-like domain (UBL), and the C-terminal ubiquitin-specific protease domain (USP).^174^ SARS-CoV-2 PLpro contains a narrow substrate-binding channel located at the interface of the thumb and palm domains^175^ (Fig. 4f). Trp106 and Asn109 are proposed to form the oxyanion hole of SARS-CoV-2 PLpro, which contributes to the stabilization of the oxyanion transition state of peptide hydrolysis.^157,173,176,177^ The protease activity of PLpro is in the monomer form, and its activity is regulated by the catalytic triad (Cys111-His272-Asp286),^178^ the zinc-binding structure located in the finger domain,^179^ and a unique gate (Leu-X-Gly-Gly) near the active center.^180^ Substrate access to the active site of PLpro is regulated by the flexible blocking loops 2 (BL2).^157,175^

Several representative inhibitors of PLpro are described here to illustrate their inhibitory mechanisms. (1) Inhibitors that target the active site, such as VIR251, a covalent peptide-mimetic inhibitor with a vinylmethylester (VME) warhead.^173^ This effectively inhibits SARS-CoV and SARS-CoV-2 PLpro activity.^173^ In the SARS-CoV-2 PLpro/VIR251 complex (Fig. 4g), P1-P4 amino acids of VIR251 insert into the S1–S4 pocket of PLpro adjacent to the active site. The GlyVME (P1) warhead forms a thioether bond with catalytic Cys111 there, and a number of polar and hydrogen bonds (engaged by the P1–P3 positions) and hydrophobic interactions (engaged by the P4 position) mediate the stability of the complex.^173^ (2) Inhibitors that target the USP domain, such as GRL-0617, a naphthalene-based non-covalent inhibitor.^151,180^ In the SARS-CoV-2 PLpro^C111S^/GRL-0617 complex (Fig. 4h), GRL-0617 resides in a cleft distant from the catalytic triad with a minimum distance of 7.5 Å to S111, and the side chains of Y268/Q269 in the BL2 loop undergo a large shift to better accommodate the compound.^174^ It has been confirmed that the interaction between GRL-0617 and PLpro blocks the C-terminus binding of ISG15 to PLpro.^151,174^ (3) Inhibitors that target multiple sites. These include YM155, an imidazolium-based inhibitor of the antiapoptotic protein survivin.^175,181^ In the SARS-CoV-2 PLpro^C111S^/YM155 complex (Fig. 4i), YM155 binds to three different sites on each PLpro molecule, including the substrate-binding pocket, the ISG15 binding site, and the zinc-finger motif.^175^ The interaction between YM155 and PLpro is stabilized by interaction networks including hydrophobic interaction, π-stacking interaction and hydrogen bonding. Ultimately, YM155 affects the activity of PLpro protease and blocks C-terminus binding of ISG15 to PLpro.

### Predicted structure of Nsp4

**Fig. 5:**
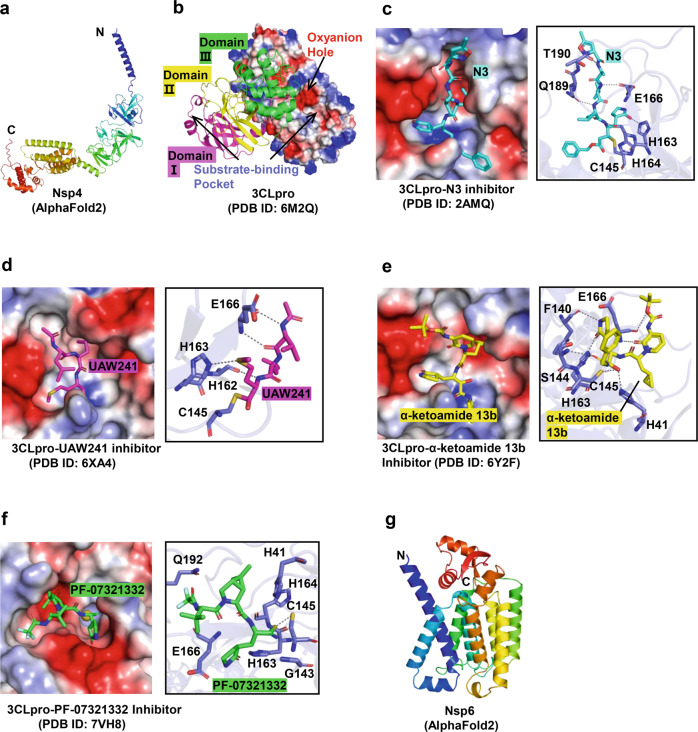
Structures of Nsp4 /Nsp5 /Nsp6 and their inhibitors. **a** Predicted structure of Nsp4 protein. **b** Overall structure of 3CLpro protein with electrostatic surface and cartoon models. **c**–**f** Structures of 3CLpro in complex with N3, calpain inhibitor II (UAW241), α-ketoamide 13b inhibitors, and PF-07321332, respectively. **g** Predicted structure of Nsp6 protein

SARS-CoV-2 Nsp4 has four transmembrane domains (TMD1~4) and a large luminal domain in the endoplasmic reticulum (ER) between TMD1 and TMD2 and a smaller luminal domain in the ER lumen between TMD3 and TMD4.^182^ Nsp4 can cause visible changes to ER structure.^183^ So far, only the crystal structure of C-terminal domain of Nsp4 (Ct-Nsp4) is available. Eribulin and Suvorexant were as promising drug candidates to target Ct-Nsp4 by screening the 1600 FDA-approved drugs using molecular docking.^184^ In this manuscript, the structure of full Nsp4 was predicted by the latest prediction tool Alphafold 2. As shown in Fig. 5a, the structure of full Nsp4 consists of nine α-helix and several β-sheets.

### Nsp5 protein

Nsp5 is also known as the main-protease (Mpro) or 3C-like protease (3CLpro). It hydrolyzes viral polyprotein 1ab, recognizing more than eleven cleavage sites to produce Nsp4-Nsp16,^185^ thus playing an essential role in viral replication and the maturation of nonstructural proteins. The absence of a human homolog of 3CLpro makes this protein one of the most attractive drug targets.^186^ Moreover, 3CLpro is highly conserved among coronaviruses;^187^ summarizing the structural similarities between viruses will be conducive to the development of broad-spectrum antiviral reagents.

The structure of 3CLpro is a homodimer, which is required for its enzymatic activity. Each protomer contains an N-finger and chymotrypsin-like domain I, domain II, and domain III^187^ (Fig. 5b). Domain I and domain II form an antiparallel β-barrel, and domain III assembles into a globular shape consisting of five α-helices. Domain III and the N-finger (residues 1–7) are involved in the dimerization of Mpro. 3CLpro has two substrate-binding sites, an oxyanion hole (composed of Gly143, Ser144, and Cys145), and a substrate-binding pocket. The latter consists of several subsites, including the deeply buried subsites s1 (F140, L141, N142, H163, and E166) and s2 (M49, Y54, H164, D187, and R188); a hydrophobic subsite known as s4 (including M165, L167, Q189, T190, and Q192); and several extended solvent-accessible subsites, s3 (E166), s5 (including T190, A191, and Q192), and s1’ (H41, G143, S144, and C145). The catalytic dyad (Cys145-His41) is located in the s1’ subsite,^188^ in which the cysteine acts as a nucleophile and the histidine as a base and proton acceptor.^187^ Based on the Schechter-Berger nomenclature, the residue sites of protease substrates or inhibitors that bind to the subsites of 3CLpro are defined as Pn, P1, P1’…Pn’. The residues at the Pn positions may vary between different coronaviruses. For example, the P1 position is often Gln, whereas the P4 position may be Tyr, Thr, Ser, Ala, or Pro.^185,189^

3CLpro inhibitors can be divided into two groups: peptide-mimetic inhibitors and non-peptide small molecular inhibitors.^189^ The former is similar to the natural peptide substrate of 3CLpro, and it is also the most studied at present. A range of specific chemical warheads, such as Michael acceptors, aldehydes, and epoxy ketones have been introduced at the P1/P2/P3 position by specific modifications of residues to strengthen the inhibitory effect on protease activity, antiviral ability, plasma half-life, and solubility.^189^ Generally, these inhibitors have covalent bond interactions with cysteine in the catalytic dyad and form a covalent adduct, which results in covalent inhibition of 3CLpro. Several representative inhibitors and their mechanisms of action are discussed below.

(1) N3 is a broad-spectrum antiviral peptidyl inhibitor with a Michael acceptor warhead.^190^ In the 3CLpro/N3 complex (Fig. 5c), N3 is embedded in the substrate-binding pocket of 3CLpro in the form of an antiparallel sheet.^186^ It not only forms covalent bonds with C^β^ and S^γ^ of C145, but also forms various non-covalent bonds with residues in the substrate-binding pocket (including H163, H164, E166, Q189, and T190).^186,189^ (2) Calpain inhibitor II is a peptidyl inhibitor with an aldehyde warhead.^189^ In the 3CLpro/Calpain inhibitor II (UAW241) complex (Fig. 5d), the P1-Met of Calpain inhibitor II forms a weak hydrogen bond with His163, occupying the s1 subsite.^189,191^ And other interactions occur between UAW241 and H162, E166, and C145. (3) α-ketoamide 13b is a broad-spectrum peptide-mimetic inhibitor of 3CLpro.^192^ In the 3CLpro/α-ketoamide complex (Fig. 5e), the α-keto warhead forms two covalent bonds with the Cys145-His41 catalytic dyad.^187^ This is distinct from other peptidyl inhibitors, which only have one covalent bond interaction with the catalytic center.^187,189^ (4) Oral antiviral PAXLOVID (Pfizer) is a mix of the antiviral PF-07321332 combined with a low concentration of Ritonavir which is an antiretroviral typically used against HIV, and have been shown to significantly reduce COVID-19 deaths in clinical trials.^193^ In the 3CLpro/PF-07321332 complex (Fig. 5f), the P1’ nitrile warhead of PF-07321332 forms a reversible covalent thioimidate adduct with Cys145 in the Cys145-His41 catalytic dyad, and therefore occupies the oxyanion hole of 3CLpro.^194^ Certainly, other groups of PF-07321332, including lactam ring, DMCP, and trifluoroacetyl group also participate in the stability of the complex. Small molecule inhibitors of 3CLpro are generally identified with a high-throughput screening of small molecule libraries, and their inhibitory mechanisms are slightly different from peptidyl inhibitors. An example is the benzotriazole-based inhibitor ribavirin,^191,195^ which is a covalent inhibitor.

### Predicted structure of Nsp6

SARS-CoV-2 Nsp6 is a transmembrane protein and possesses eight transmembrane domains. Nsp6 expression inhibited the formation of a hybrid pre-autophagosomal structure (HyPAS).^196^ Moreover, Nsp6 can strongly blocked MAVS (mitochondrial antiviral signaling protein)-induced interferon β production and binds TANK binding kinase1 (TBK1) to suppress interferon regulatory factor 3 (IRF3).^197,198^ Nsp6 also interacts with the sigma-1 receptor, which is considered an effective candidate host protein for host-based repurposing approaches to treat COVID-19 patients.^199^ So far, there is no available structural information for Nsp6. The predicted overall three-dimensional structure of NSP6 consists of 14 α-helices, a C-terminal, two antiparallel β-strands, and 16 turns (Fig. 5g). The agonist of sigma receptors dextromethorphan binding leads to overall destabilization of Nsp6.^200^

### Nsp7 and Nsp8 protein

Nsp7, Nsp8, and Nsp12 are the core components of the coronavirus replication machinery, in which Nsp12 acts as the RNA-dependent RNA polymerase (RdRp) and Nsp7/Nsp8 function as cofactors of Nsp12, possess primase activity,^201,202^ and mediate RdRp activity.^203^ Nsp7 and Nsp8 are highly conserved among coronaviruses,^204,205^ and further study of their structure and function will be conducive to the development of broad-spectrum inhibitors.

**Fig. 6:**
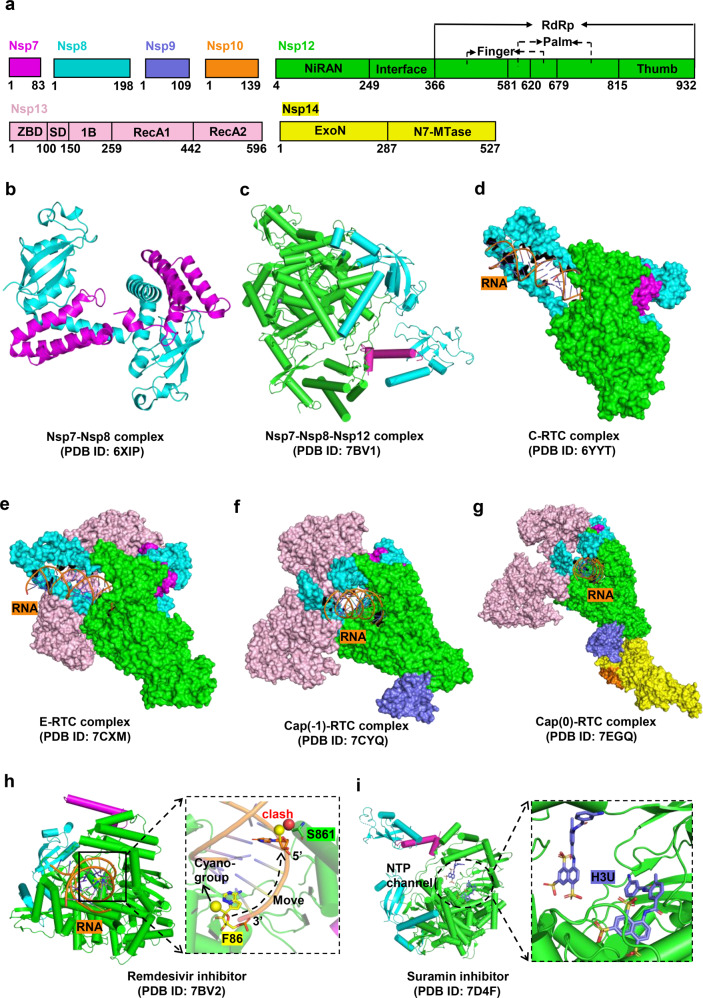
Structures of SARS-CoV-2 RTC complexes and cognate inhibitors. **a** Schematic representations of Nsp7, Nsp8, Nsp9, Nsp10, Nsp12, Nsp13, and Nsp14 proteins organizations. **b**–**g** Structures of Nsp7-Nsp8, Nsp7-Nsp8-Nsp12, the central RTC (C-RTC, Nsp12-Nsp7- Nsp8), the elongation RTC (E-RTC, Nsp12-Nsp7-Nsp8-Nsp13), cap (−1)-RTC (Nsp12-Nsp7-Nsp8-Nsp13-Nsp9), and cap (0)-RTC (Nsp12-Nsp7-Nsp8- Nsp13-Nsp9-Nsp14-Nsp10) complexes, respectively. **h, i** Structures of C-RTC with bound inhibitors Remdesivir (F86) can bind to the RNA strand. The schematic of the inhibition state is shown on the right (**h**). Two suramin (H3U) molecules occupy the catalytic cavity (**i**)

Nsp7 consists of 83 amino acids with only four α-helices (α1-α4) comprising its structure.^204,205^ In contrast to the relatively stable structure of α2/α3, α1/α4 adapts different lengths, positions, and relative orientations in various environments, and this variability plays an essential role in the assembly of the Nsp7-Nsp8 complex.^205^ Nsp8 consists of 198 amino acids, including a long helical N-terminal domain (residues 1 to 77) and a conserved C-terminal domain (residues 78–198) (Fig. 6a). The N-terminal domain of Nsp8 is highly flexible and prone to proteolysis during the process of crystallization.^204,206^ Another feature of this domain is a positive electrostatic surface that may be used to bind viral RNA.^207,208^ The C-terminus of Nsp8 comprises five α-helices and one four-stranded antiparallel β-sheet, and its topology resembles a golf club.^209^ The SARS-CoV-2 Nsp7-Nsp8 complex forms a hetero-tetramer [PDB ID 7JLT and 7DCD]^204,206^ (Fig. 6b), in contrast to the hexadecameric structure of SARS-CoV Nsp7-Nsp8.^210^ Two types of interfaces exist in the Nsp7-Nsp8 complex that synergistically mediates the stability of dimerization and tetramerization.^204^ Notably, both interfaces also mediate the dynamic assembly of the Nsp7-Nsp8-Nsp12 complex, and a large proportion of the residues located on the two interfaces are highly conserved among coronaviruses.^204^

The structural details of the Nsp7-Nsp8 complex reveal possibilities for the development of allosteric inhibitors that specifically inhibit RdRp activity.^204^ Distinct from nucleotide analogs that directly target RdRp, allosteric inhibitors interfere with the activity of RdRp machinery by disrupting the assembly of Nsp7-Nsp8-Nsp12. For example, the Nsp7^N37V^ mutant, which affects the stability and activity of Nsp7-Nsp8-Nsp12 complex, could be exogenously introduced into the infected cells to inhibit viral proliferation.^204^

### Nsp9 protein

SARS-CoV-2 Nsp9 shares 97% sequence identity with that of SARS-CoV Nsp9, both of which belong to the oligonucleotide/oligosaccharide binding superfamily (OB-fold) and specifically bind to single-stranded DNA and RNA oligonucleotides.^211^ Nsp9 is involved in the formation of the replication and transcription complex (RTC) and plays a significant role in viral replication.^190,207,212^ The crystal structure of SARS-CoV-2 Nsp9 is a homodimer,^213^ and the arrangement of protomers is conserved with respect to other coronaviruses. The core comprises a seven β-strand enclosed β-barrel, and the C-terminus has a flexible α-helix (residues 96–109) that contains the conserved protein–protein interaction motif (GXXXG). Two combination modes, the helix interface and the sheet interface, contribute to the stability of the dimer. The former is formed by two GXXXG motifs with strong van der Waals interactions, whereas the latter includes β5 and connection loops.^214,215^

The mechanism of RNA binding with Nsp9 is still unclear. A previous study identified the possible RNA-binding sites (Phe40, Val41, and Ile91) by observing the chemical perturbation of Nsp9 titrated with ssRNA using nuclear magnetic resonance (NMR) titration assays.^216^ Subsequently, targeted substrates that bind to these sites (or adjacent to these sites) were screened from a small fragment library; the uracil-analog FR6, which has a weak affinity with Nsp9, was obtained.^216^ In the Nsp9/FR6 complex, a tetrameric π–π stacking between the pyrimidinedione ring of FR6 and the aromatic ring of Phe40 induces a hexameric form of Nsp9 (a “trimer of dimers”).^216^ Changes in the oligomerization state may alter RNA entry channels and thus affect RNA binding. In addition to the inhibitors of Nsp9-RNA binding described above, other inhibitors have been identified that target the Nsp9 GXXXG motif to disrupt the dimer interface, affecting RNA binding and viral proliferation.^213,217^ Finally, several potential inhibitors (such as imidazolium salts^218^ and teicoplanin^219^) have also been identified through molecular docking, but the inhibition mechanisms are unknown.

### Nsp10 protein

Nsp10, a protein unique to viruses,^125^ plays a crucial role in viral mRNA capping via methylation, a process that promotes stability and effective translation of viral RNA.^220,221^ Four sequential enzymatic reactions are involved in coronavirus RNA capping. Initially, RNA 5′-triphosphatase in Nsp13 hydrolyzes nascent RNA to produce pp-RNA; an unknown guanylyl-transferase (GTPase, possibly Nsp12)^222^ then hydrolyzes GTP and transfers the product (GMP) to pp-RNA to form Gppp-RNA; in the subsequent reaction, N7-methyltransferse (N7-MTase) methylates Gppp-RNA to create Cap-0 (m7GpppA_1_); in the final step, the ribose 2′-O of the first nucleotide in Cap-0 is methylated by Nsp16, resulting in the formation of Cap-1 (m7GpppA_1m_).^223,224^ Throughout the process, Nsp10 specifically binds to and stimulates the N7-MTase and the 2′-O-methlytransferase (2′-O-MTase) activity of Nsp14 and Nsp16, respectively,^223,224^ which provides the molecular connector between proofreading and capping activities.^225^ Therefore, a scheme is proposed to interfere with the activity of Nsp14 and Nsp16 with the peptide derivatives of Nsp10.^226,227^ For example, a peptide (defined as K29) from SARS-CoV Nsp10 (resides 68 to 96) could significantly inhibit the activity of Nsp16.^226^ The same approach could be applied to screen peptide inhibitors against SARS-CoV-2. In this section, we focus on the structure of Nsp10; the structure of the Nsp10-Nsp14 and Nsp10-Nsp16 complexes will be described in the following chapters.

SARS-CoV-2 Nsp10 and SARS-CoV Nsp10 have nearly identical sequences (99%) and structures,^125^ both of which comprise five α-helices, an antiparallel β-sheet, and two zinc-finger structures (ZnF1 and ZnF2) (PDB ID 6ZPE).^125^ ZnF1 is coordinated by three cysteines and one histidine, whereas ZnF2 is coordinated by four cysteines. The zinc-finger structure enables Nsp10 to play a significant role in viral RNA synthesis.^228^

### Nsp12 protein

Coronaviruses utilize the RTC to complete viral genome replication and mRNA transcription.^9^ Nsp12, which is highly conserved among coronaviruses, is the core component of RTC. Nsp12 assembles with other nonstructural proteins to form various architectures^229^ such as the central RTC (C-RTC, Nsp12-Nsp7-Nsp8)^230^ (Fig. 6c, d), the elongation RTC (E-RTC, Nsp12-Nsp7-Nsp8-Nsp13)^222^ (Fig. 6e), the Cap (−1)′-RTC (Nsp12-Nsp7-Nsp8-Nsp13-Nsp9)^222^ (Fig. 6f), the Cap (0)-RTC (Nsp12-Nsp7-Nsp8-Nsp13-Nsp9-Nsp14-Nsp10)^231^ (Fig. 6g) and the Cap (1)-RTC (Nsp12-Nsp7-Nsp8-Nsp9-Nsp16-Nsp10). These architectures play a pivotal role in viral proliferation and host immune regulation. Therefore, further understanding of the structure and catalytic mechanism of Nsp12 could accelerate the development of broad-spectrum antiviral agents.

SARS-CoV-2 Nsp12 is composed of 932 amino acids, consisting of the N-terminal nidovirus RdRp-associated nucleotidyl-transferase domain (RiRAN, residues 117–250),^232^ the interface domain (residues 251–365), and the RdRp domain. The latter is subdivided into the finger subdomain (residues 367–581 and 621–679), the palm subdomain (residues 582–620 and 680–815), and the thumb subdomain (residues 816–932).^233^ The single RdRp domain folds into a right-hand shape that resembles other RNA polymerases,^233^ whereas the heterotrimeric complex formed by Nsp12, Nsp7, and Nsp8 (PDB ID 6YYT and 7BV2) is packed into a stable closed conformation.^203,234^ Two conserved zinc-finger structures in this complex (ZnF1 and ZnF2, coordinated by H295/C301/C306/C310 and C487/H642/C645/C646) contribute to the structural integrity of RdRp.^234^ Seven conserved active sites (motifs A-G) are located throughout the RdRp domain, in the palm (motifs A, B, C, D, E) and finger (motifs F and G) subdomains, which direct and stabilize the RNA template-product duplex by interacting with RNA template- and primer-strands.^234^ In addition, there is a positively charged ‘sliding poles’ structure formed by two copies of Nsp8 N-terminal domain in the complex, which is verified to accommodate the RNA duplex outside the active sites.^203^

Presently, numerous nucleoside analog drugs are readily available, such as ribavirin and favipiravir (guanine analogs),^235,236^ sofosbuvir (uridine analog),^237^ and molnupiravir (also named MK-4482 or EIDD-2801),^238^ remdesivir, and galidesivir (adenosine analogs).^239^ Nsp12 is considered a primary target for these.^240^ For example, remdesivir is a broad-spectrum antiviral agent and the first FDA-approved drug for the treatment of COVID-19.^241,242^ In the C-RTC/RNA/remdesivir complex (Fig. 6h), the remdesivir-monophosphate (RMP) is located at the catalytic active site of RdRp and covalently incorporated into the RNA primer strand at the +1 position.^234^ The RMP interacts with Nsp12 in a manner identical to adenosine monophosphate (AMP), but the steric clash between the cyano group of RMP and Ser861 of Nsp12 blocks RNA translocation after incorporation of three bases following RMP.^234^ This ultimately results in delayed chain termination.^243,244^ In addition to the above mechanism, another inhibition forms of remdesivir action was recently proposed, namely RNA template-dependent inhibition of RdRp.^245^ A promising orally available drug to treat COVID-19, molnupiravir is receiving more and more attention. The inhibition mechanism of RdRp by molnupiravir is different from remdesivir. In the process of RNA synthesis, the active form of molnupiravir, β-D-N^4^-hydroxycytidine (NHC) triphosphate (MTP), can form stable base pair with either G or A in the RdRp active center.^246^ Notably, these mis-incorporations would not lead to stalls RdRp like remdesivir, and this NHC-containing RNA product can also be used as an RNA template in a new round of RNA synthesis.^246^ So, molnupiravir can adopt a two-step mutagenesis mechanism to cause an “error catastrophe” during viral RNA replication. Certainly, the difference between molnupiravir and remdesivir in inhibiting RNA synthesis by RdRp also indicates that nucleoside analogs have diverse antiviral mechanisms.

There are several other drugs that target RdRp, such as suramin,^247^ a poly-sulfonated trypan blue derivative that effectively inhibits a variety of viruses including SARS-CoV-2.^26^ In the RdRp-suramin complex (Fig. 6i), two suramin molecules occupy the catalytic cavity, which in turn blocks the RNA template-primer strand from binding to the active site and blocks NTP substrate entry into the catalytic site.^248^ Nsp12 additionally has GTPase activity to catalyze the formation of GpppA, but Nsp9 specifically inhibits this activity.^222^ In the Nsp12/Nsp7/Nsp8/Nsp13/Nsp9 complex (PDB ID 7CYQ), the N-terminus of Nsp9 inserts into the catalytic center of Nsp12, interacting with the bound GDP.^222^ Therefore, peptides with similar properties to the N-terminus of Nsp9 could be designed to inhibit the activity of Nsp12. Guanine analogs can also be utilized to inhibit the GTPase activity of Nsp12 and interfere with mRNA capping.^222^

### Nsp13 protein

Nsp13 has helicase and RNA 5′-triphosphatase activity, with which it participates in unwinding DNA or RNA during RNA replication in an ATP-dependent manner^249,250^ and in the first step of mRNA capping,^251,252^ respectively. Nsp13, therefore, has crucial roles in viral proliferation and can be regarded as a drug target for treating COVID-19.^253^

SARS-CoV-2 Nsp13 consists of 601 amino acids, including the N-terminal zinc-binding domain (ZBD) (residues 1–100), a stalk domain (SD, residues 101–150), an inserted domain 1B (residues 151–259), and two helicase domains: RecA1 (residues 260–442) and RecA2 (residues 443–596).^254,255^ Nsp13 and the C-RTC (Nsp12-Nsp7-Nsp8) form a stable complex, E-RTC (L) (Fig. 6e), and the helicase activity of Nsp13 is enhanced by the formation of this complex.^207,208,256^ Two Nsp13 protomers play different roles in the E-RTC complex (PDB ID 7CXM and 7CXN);^208^ Nsp13-1 stabilizes the structure of RTC by interacting with Nsp12 and Nsp8-1, whereas Nsp13-2 (with a larger conformational shift compared to the free state) provides an RNA-binding channel for the unpaired 5′ extension of RNA template.^222^ Residues in the Nsp13-2 RNA-binding channel that are involved in RNA recognition are highly conserved in coronaviruses. These include N361 in the RecA1 domain, S468/T532/D534 in the RecA2 domain, and R178/H230 in the 1B domain.^222,257^ The paired portion of template-primer RNA is located in the pocket formed by Nsp8 and Nsp12. In the process of viral RNA replication, Nsp13 anchors the 5′ extension of template RNA and stimulates the RdRp backtracking that is a ubiquitous transcriptional regulatory mechanism.^207,257–260^ A 3′ end of primer-strand RNA containing mismatched nucleotides can be guided toward the RdRp NTP entry tunnel as a result of the RdRp backtracking capability, providing access for proofreading machinery (Nsp14/Nsp10).^257,261^ This action can allow nucleotide analog-based resistance.^262^ Therefore, inhibitors that interfere with the activity of Nsp13 and RdRp backtracking should be administered in combination with nucleoside analogs to prevent drug resistance.

### Nsp14 protein

As a dual-functional enzyme, Nsp14 has both 3′-to-5′ exoribonuclease (ExoN) and N7-MTase activities, which are responsible for nascent RNA proofreading and mRNA capping during viral RNA replication, respectively.^224,263,264^ The significance of Nsp14 in high-fidelity replication of viral RNA and host immune defense escape makes this protein an attractive target for antiviral treatment.^265–267^ Furthermore, Nsp14 is highly conserved among the coronaviruses,^268^ and drugs targeting this protein have the potential to be pan-inhibitors for other viruses.

SARS-CoV-2 Nsp14 consists of 527 amino acids, including the N-terminal ExoN domain (residues 1–287) and the C-terminal N7-MTase domain (residues 288–527). The activity of ExoN can be stimulated by the cofactor Nsp10 independent of the N7-MTase domain.^264,269,270^ When ExoN forms the ExoN-Nsp10 complex, Nsp10 does not undergo a significant conformational change,^270^ and the complex structure resembles the Asp-Glu-Asp-Asp (DEDD)-type exonuclease.^264,270^ Both possess typical DED/Edh motifs (D90/E92/E191/H268/D273)^271^ and adopt similar topological folds (a central twisted β-sheet flanked by α-helices on either side). However, distinct from the DEDD-type exonuclease, Nsp14 contains two zinc-binding sites (ZnF1 and ZnF2) that are located on both sides of the β-sheets and coordinated by C207/C210/C226/H229 and H257/C261/H264/C279, respectively. These zinc-finger structures are associated with the stability and enzymatic activity of ExoN.^264^ A convoluted loop (residues 288–301) joins the N7-MTase domain and the ExoN domain. In contrast to ExoN, N7-MTase activity is independent of Nsp14-Nsp10 complex formation,^272,273^ and there are no interactions between the N7-MTase domain and Nsp10. The N7-MTase domain adopts a noncanonical MTase fold with a three-stranded β-sheet insertion and a peripheral zinc finger (ZnF3). ZnF3, however, has a limited role in the activity of N7-MTase, and may instead be involved in protein–protein interactions.^274^

**Fig. 7:**
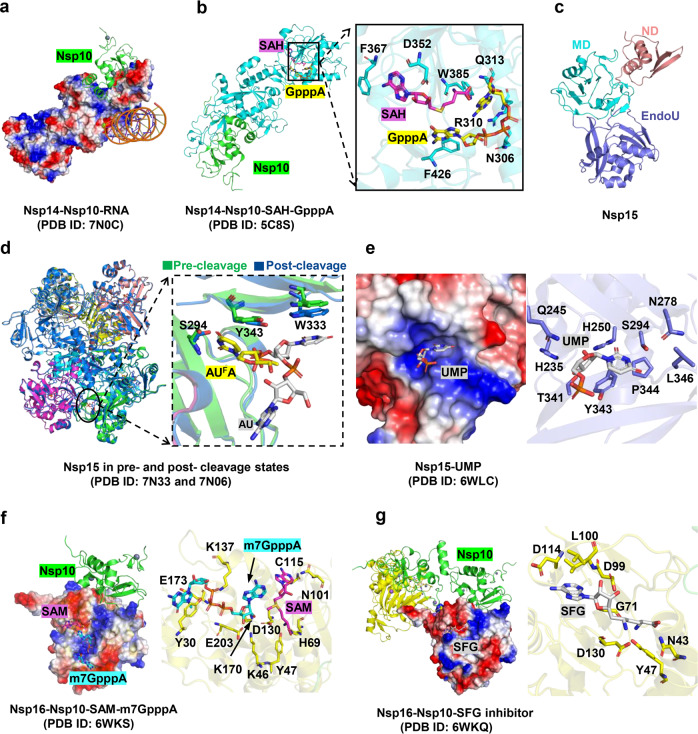
Structures of Nsp14 /Nsp15/Nsp16 and their inhibitors. **a** Cryo EM structure of the SARS-CoV-2 Nsp10-Nsp-14 RNA complex. Nsp14 is illustrated with electrostatic surface. Nsp10 is illustrated with cartoon in green. **b** The structure of the Nsp14-Nsp10 in complex with functional ligands S-adenosyl-L-homocysteine (SAH) and GpppA shown in sticks. **c** The overall structural of Nsp15. **d** Conformational changes between Nsp15 in pre- and post- cleavage states. **e** Structures of Nsp15 in complex with uridine-5′-monophosphate (UMP). **f** The structure of SARS-CoV-2 Nsp16-Nsp10 in complex with RNA cap analogue (m7GpppA) and S-adenosyl methionine (SAM). The m7GpppA and SAM are shown in sticks. Nsp16 is indicated as electrostatic surface. **g** The structure of Nsp16-Nsp10 heterodimer in complex with sinefungin (SFG). SFG is shown in sticks. Nsp16 is illustrated with electrostatic surface and cartoon in yellow

Understanding the molecular mechanisms of proofreading and methylation will be beneficial in developing inhibitors of Nsp14. In the ternary complex of Nsp14-Nsp10-RNA (bearing a 3′-end mismatch) (Fig. 7a), only three base pairs (including a mismatched C-U pair at the 3′-end of the RNA) are located in a narrow pocket on the ExoN surface, and most of the RNA helix is in an unbounded or solvent-exposed state.^275^ This complex of RNA substrate and Nsp14 is determined by two key residues (H95 and P142), which restrict the depth of the substrate-binding pocket and may force the separation of primer-strand and template-strand RNA.^275^ There are also two Mg-binding sites (Mg1 and Mg2) in the ternary complex, in contrast to the one Mg-binding site in the binary structure of Nsp14-Nsp10.^264,270^ One magnesium ion activates a water molecule for nucleophilic attack and the other stabilizes the O3′ leaving group of -1C_P_.^275^ The methylation reaction mechanism of the N7-MTase can be determined from the structure of the Nsp14-Nsp10-SAH-G_PPP_A complex, in which the binding site of the substrate S-adenosyl methionine (SAM) is adjacent to the GpppA binding site (Fig. 7b). This combination may facilitate methyl transfer from donor to acceptor.^264,274^

As an essential exoribonuclease in coronavirus, Nsp14 removes both mis-incorporated nucleotides and nucleotide analogs from the nascent RNA, making viruses that encode it prone to develop nucleotide analog-based antiviral resistance.^276,277^ The combination of Nsp14 inhibitors and nucleotide analogs (such as remdesivir,^278^ sofosbuvir,^279^ and ribavirin) have the potential to resolve this issue.^280^ Nsp14 inhibitors take multiple forms, as described below.

(1) 3′-deoxy nucleotide analogs inhibit the activity of ExoN.^275^ (2) Zn^2+^-ejecting agents, such as disulfiram and ebselen, synergistically inhibit Nsp14 activity via their three zinc-binding sites.^281,282^ (3) SAM competitive inhibitors and SAM analogs, such as S-adenosyl-homocysteine (SAH), sinefungin (SFG), and aurintricarboxylic acid (ATA)^273,283^ interfere with N7-Mase activity and subsequently impede 5′-end cap formation.^284^ In addition to these inhibitors, mutations in Nsp14 can lead to virus attenuation and induction of higher interferon response. Live attenuated virus vaccine development is, therefore, an option for this target in addition to the development of antibodies.^285–287^

### Nsp15 protein

Nsp15 (also called EndoU)^288^ is a uridine-specific, Mn^2+^-dependent endoribonuclease that has functional characteristics and active sites similar to eukaryotic RNase.^289,290^ Coronavirus Nsp15 cleaves the 5′-polyuridine tracts in negative-strand RNA and prevents the activation of host pattern recognition receptor MDA5-mediated immune response.^291–293^ The crucial function and extreme conservation of Nsp15 in coronaviruses make it a promising target for COVID-19 treatment.^294^

SARS-CoV-2 Nsp15 consists of 347 amino acids, including the N-terminal domain (ND, residues 1–64), the middle domain (residues 65–182), and the C-terminal EndoU domain (residues 207–347).^295^ The overall structure of SARS-CoV-2 Nsp15 is a homo-hexamer with D3 symmetry^289,295–297^ (Fig. 7c). Several conserved residues in Nsp15 (including His235, His250, Lys290, Thr341, Trp333, Tyr343, Ser294, Gly248, Lys345, Val295, and Gln245) are involved in substrate specificity, nuclease activity, and oligomerization of EndoU.^295,298^ His235, His250, and Lys290 constitute the catalytic triad and utilize the general acid-base catalytic mechanism to complete the cleavage reaction.^289,295–297^ The EndoU domain has conformational variability, and the substrate uridine-5′-Monophosphate (UMP) restrains this dynamic.^295^ Notably, allosteric regulation exists in the excision reaction of EndoU,^297,299^ and the base binding sites are different between RNA in the pre- and post-cleavage states^297^ (Fig. 7d, e). In the Nsp15/5′-UMP and Nsp15/AU^F^A complexes Ser294 forms two hydrogen bonds with O2 and N3 of the uracil base and Tyr343 forms π–π stacking interactions with the ribose ring.^289,300^ Consequently, Ser294 and Tyr343 of Nsp15 may be responsible for base discrimination by EndoU.^295,300^ In the Nsp15/AU-3′P complex (PDB 7N06), Trp333 forms π–π stacking interactions with uracil, but Ser294 no longer forms hydrogen bonds with uracil due to a conformational flip.^297^ The ND domain also plays an indispensable role in the function of Nsp15, participating in protein oligomerization and RNA binding.^297,299^

Some inhibitors targeting Nsp15 are uracil derivatives or modified oligonucleotides containing these derivatives, such as tipiracil or modified RNA with 2′-fluorine instead of 2′-OH on the uridine ribose.^289,301^ Tipiracil effectively inhibits the activity of EndoU and binds in a manner similar to uridine in the Nsp15/tipiracil complex.^289,302^ Compounds that disturb the stability of the hexamer conformation, and thus interfere with the activity of EndoU, could be screened as inhibitors of Nsp15. An attenuated live virus vaccine with Nsp15-defective SARS-CoV-2 has also been proposed.^292^

### Nsp16 protein

As mentioned above, Nsp16 is a Mg^2+^ and Nsp10-dependent 2′-O-methyltransferase in coronaviruses that methylates the ribose 2′-O of the first nucleotide in Cap-0 mRNA (m7G_0ppp_A_1_-RNA) to produce Cap-1 mRNA (m7G_0ppp_A_1m_-RNA).^303–306^ In the Nsp16-Nsp10-SAM-m7G_0_pppA_1_ (or m7G_0_pppA_1m_) complex (Fig. 7f), an Nsp16 protomer is anchored on the top of an Nsp10 promoter.^307–309^ The structure of Nsp10 in the heterodimer does not undergo a significant conformational change, aside from the α1-helix, as previously reported.^125,307,308^ Nsp16 employs a Rossmann-like fold consisting of a centrally located twisted β-sheet of eight β-strands (β1-β8-β9-β6-β7-β2-β3-β4-β5) flanked by α-helices and β-strands. Various substrates of Nsp16 bind in different pockets on the surface of the protein. Cap is located in a positively charged pocket with intrinsic plasticity, in which Lys137 and Tyr30 form a partial enclosure to restrict the movement of Cap.^308^ SAM is located in a negatively charged pocket, and a number of interactions (including electrostatic, hydrophobic, and Van der Waals interactions) mediate the stability of SAM; Nsp10 also plays a fixed role in the SAM-binding pocket.^272,306^ The binding pockets of SAM and Cap are separated by a four amino-acid long stretch (residues 131–134). Ligand-binding sites with unclear functions have also been found on the surface of Nsp16. For example, a positively charged pocket close to the Cap binding pocket, possibly used to bind longer RNA, has been identified;^307,309^ on the back of the conservative catalytic center (Lys46-Asp130-Lys170-Glu203),^310,311^ there is another ligand-binding pocket that accommodates adenosine and other small molecules with a heterocyclic ring.^272,307^ The interface of Nsp16-Nsp10, composed of multiple hydrophobic interactions and hydrogen bonds, largely overlaps with the interface of Nsp14-Nsp10.^308^ A key difference between the two interfaces lies in the α1-helix of Nsp10. Compared with the apo structure of Nsp10, the Nsp10 α1-helix retains the same conformation in the Nsp14-Nsp10 complex, whereas there is an obvious wiggle (~130°) in the Nsp16 and Nsp10 complex.^270^

The Nsp16/Nsp10 complex in SARS-CoV-2 has high homology with those in other coronaviruses, and most residues that participate in catalysis and Cap/SAM binding are conserved.^307,312^ Therefore, inhibitors that target this protein are promising candidates for development into broad-spectrum antiviral agents. Inhibitor design for Nsp16 can be viewed from multiple perspectives. SAM analogs (such as sinefungin [SFG], a pan-inhibitor of MTase) can be utilized to occupy the SAM-binding pocket of Nsp16, inhibiting the activity of 2′-O-methyltransferase.^308^ In the Nsp16-Nsp10-SFG complex (Fig. 7g), SFG has the same interactions with residues of Nsp16 as SAM does.^308^ Another approach is related to the protein conformation of Nsp16, which varies significantly before and after Cap is bound. If small molecules that stabilize the protein conformation can be identified, it may effectively inhibit the methylation of viral mRNA.^308^ The specific ligand-binding pocket with unknown function in Nsp16 is also a potential target for inhibitor binding.^307^ Inhibitors that disrupt the interaction between Nsp16 and Nsp10 can also be applied, such as the peptide inhibitor mentioned above.^226,227^ Finally, the construction of a live attenuated virus vaccine with Nsp16-defective SARS-CoV-2 may be possible.^286^

## Accessory proteins of SARS-CoV-2

Accessory protein genes are interspaced between or within the viral structural protein genes, and have some genus or species specificity. SARS-CoV-2 encodes nine accessory proteins, including ORF3a (275 aa), ORF3b (22 aa), ORF6 (61 aa), ORF7a (121 aa), ORF7b (43 aa), ORF8 (121 aa), ORF9b (97 aa), ORF9c, and ORF10 (38 aa).^313^ These accessory proteins play essential roles in virus-host interactions,^314^ affecting host innate immunity, autophagy, and apoptosis, as well as viral proliferation and virulence.^315–317^ Therefore, targeting these accessory proteins to interfere with virus-host interactions is also a novel approach for treating COVID-19. The structure of the four accessory proteins (ORF3a, ORF7a, ORF8, and ORF9b) that have been reported so far are described below.

### ORF3a protein

ORF3a is highly conserved between SARS-CoV-2 and SARS-CoV (72.7% sequence identity),^318^ and is closely related to viral pathogenicity by disrupting the cellular physiology of the host cell.^319,320^ It not only promotes cytokine storms by activating NF-κB signaling and NLRP3 inflammasomes,^321^ but also regulates host cell apoptosis and autophagy.^322,323^ Furthermore, ORF3a is another viroporin in addition to the envelope (E) protein,^324^ which participates in the release of viral particles as an ion channel.^325^

**Fig. 8:**
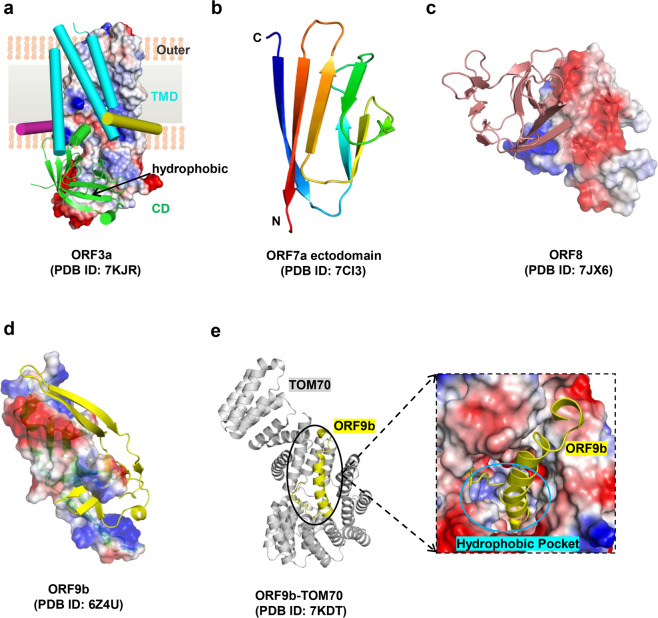
Structures of accessory proteins. **a**–**d** Overall structure of ORF3a, ORF7a, ORF8, and ORF 9b, respectively. **e** Structure of ORF9b in complex with human protein TOM70, a subunit of the mitochondrial import receptor. ORF9b is shown as yellow cartoon. TOM70 is illustrated with electrostatic surface on the right. The ORF9b binds to the hydrophobic pocket of TOM70 and occupies its binding site

ORF3a is the largest accessory protein in SARS-CoV-2.^319^ The crystal structure is an oligomer including a dimer and tetramer (PDB ID 7KJR), and the tetramer conformation is formed by two dimers^326^ (Fig. 8a). A pair of short α-helices divide the dimer structure into the transmembrane domain (TMD) that forms a central channel and the cytosolic domain (CD) located in the cytosol. The TM domain consists of three transmembrane α-helices and the CD domain forms a β-barrel comprising eight antiparallel β-strands. The stability of the dimer is dependent on multiple non-covalent interactions (π–π stacking and hydrophobic interactions) between two CD domains. In addition, several functional domains are also present in ORF3a, such as a cysteine-rich pocket that facilitates oligomerization and a tyrosine-based sorting motif (YXXφ) that mediates intracellular and extracellular transport.^313,326–328^

ORF3a binds to host heme oxygenase HMOX1,^329^ which plays an essential role in anti-inflammatory effects via the NLRPS pathway.^330–332^ Treatment with compounds that inhibit the interaction between ORF3a and HMOX1 is an effective strategy for the treatment of COVID-19. Additionally, anti-ORF3a antibodies have been found in the plasma of convalescent COVID-19 patients.^333,334^

### ORF7a protein

SARS-CoV-2 ORF7a is a type-I transmembrane protein composed of an N-terminal signal peptide (residues 1–15), Ig-like ectodomain (residues 16–96), transmembrane region (residues 97–116), and ER retention motif (KRKTE) (residues 117–121).^335^ The structure of the Ig-like ectodomain is similar to the intracellular adhesion molecule-2 (ICAM-2) of the immunoglobulin superfamily,^6,336^ which is folded into a β-sandwich by seven β-strands (Fig. 8b); two pairs of intramolecular disulfide bonds (Cys23-Cys58 and Cys35-Cys67) participate in the stability of the structure.^335^

ORF7a triggers an immune response in host cells.^337^ It not only activates NF-κB signaling and induces the expression of proinflammatory cytokines and chemokines^338^ but also inhibits the induction of type-I interferon by blocking STAT2 phosphorylation.^339–341^ Therefore, ORF7a is an attractive therapeutic target for COVID-19. Notably, the C-terminus of ORF7a plays a non-negligible role in host immunomodulation, but this region has a higher mutation frequency than the Ig-like ectodomain. This variability would be important to the design of broad-spectrum antiviral agents against this protein.

### ORF8 protein

As the most variable accessory protein in β-coronaviruses, ORF8 frequently undergoes gene recombination, deletion, and substitution, which is associated with pathogenicity and adaptive evolution.^342–344^ SARS-CoV-2 ORF8 is a multifunctional protein that induces host cell apoptosis,^345^ suppresses the host innate immune response by downregulating the class I major histocompatibility complex (MHC-I)-mediated viral antigen presentation,^346^ and functions as an antagonist of type-I interferon (IFN).^131,339,347,348^ Therefore, ORF8 is considered a potential antiviral target.

SARS-CoV-2 ORF8 consists of 121 amino acids, including the N-terminal TM domain (residues 1–17) and the central Ig-like domain (residues 18–121). The TM domain is responsible for endoplasmic reticulum (ER) import and secretion, and the Ig-like domain can interact with multiple host factors involved in pulmonary inflammation and fibrogenesis.^349,350^ The crystal structure of SARS-CoV-2 ORF8 is a homodimer (PDB ID 7JTL), and each monomer comprises eight antiparallel β-strands with three pairs of intramolecular disulfide bridges between the strands^351^ (Fig. 8c). Drugs targeting ORF8 can be designed based on the structure to disrupt interactions between ORF8 and multiple host proteins, such as lysyl oxidase (LOX), interleukin 17 receptor (IL17RA), and growth/differentiation factor 15 (GDF15).^349^ Additionally, ORF8 protein elicits robust antibody responses in the host, and these antibodies have become the major serological marker of SARS-CoV-2 infection.^329,337,352^ In the future, these specific antibodies can be screened and identified.

### ORF9b

ORF9b is an accessory protein unique to SARS-CoV and SARS-CoV-2.^353^ Both SARS-CoV ORF9b and SARS-CoV-2 ORF9b proteins are dimerized and assembled into a tent-like shape by two intertwined monomers (PDB ID 2CME and 6Z4U)^354^ (Fig. 8d). The monomer consists entirely of β-strands. A number of charged residues are distributed on the surface and a hydrophobic central tunnel for lipid binding is located inside the dimer.^354^

SARS-CoV-2 ORF9b specifically binds to the mitochondrial surface receptor protein TOM70,^350^ which cooperates with molecular chaperone Hsp90 to promote the transfer of preproteins to mitochondria and activates the host antiviral immune response.^355,356^ The N-terminal tetratricopeptide repeats (TPRs) of TOM70 are associated with the Hsp90 C-terminal EEVD motif, and the C-terminal TPRs bind to the mitochondrial preprotein. In the ORF9b-TOM70 complex (PDB ID 7KDT and 7DHG) (Fig. 8e), a single ORF9b binds to the hydrophobic pocket at the C-terminal domain (CTD) of Tom70, occupying the preprotein binding site and allosterically inhibiting the interaction between Tom70 and Hsp90.^350,353^ This ultimately blocks host mitophagy and interferon signaling.^350,357^ Therefore, inhibitors that disrupt the interaction between ORF9b and Tom70 could be screened to interfere with viral proliferation. Antibodies targeted to ORF9b have also been observed in the plasma of convalescent COVID-19 patients.^358^

## Conclusion

Despite great progress in the development of antiviral drugs based on the SARS-CoV-2 protein structures, there are still some challenges in this field. Firstly, although most protein structures have been determined, the functions of SARS-CoV-2 proteins have not been fully characterized. For example, recent studies surprisingly found that Nsp12 not only employ NDP distinguishing from conventional NTP as a substrate to synthesis RNA, but also likely display both exoribonuclease and proofreading activity, and its proofreading activity increased with the combination of Nsp14-ExoN.^359^ Secondly, the molecular basis of dynamic interactions of SARS-CoV-2 with host need to be further investigated. For example, a large number of host proteins that interact with SARS-CoV-2 proteins have been identified, but the information of the complex structures of the viral proteins of SARS-CoV-2 with host proteins is still limited. Lastly, these drugs targeting a single site are particularly vulnerable to the evolution of drug resistance through random viral mutation. The therapeutic effects of structural-based drugs on the numerous emerging SARS-CoV-2 variants need to be further analyzed. Simultaneously it might be a better choice to develop drugs targeting multiple key sites or conserved binding sites of viral protein with host proteins in the life cycle of SARS-CoV-2. Currently, we have been hunted ways to abolish the virus after the variants happened, however, the most important question is how to take precautions before the pandemic variants.

In conclusion, we summary the representative protein structures of SARS-CoV-2 and the structural-based drug design utilizing these proteins and predict the structures of those proteins that lack precise structural information, including M, Nsp4 and Nsp6 by using the latest prediction tool, like Alphafold 2. The review is aimed at providing theoretical insight for mitigating the current COVID-19 pandemic and potential future coronavirus outbreaks.
